# Coordinating Cytoskeleton and Molecular Traffic in T Cell Migration, Activation, and Effector Functions

**DOI:** 10.3389/fcell.2020.591348

**Published:** 2020-10-21

**Authors:** Marta Mastrogiovanni, Marie Juzans, Andrés Alcover, Vincenzo Di Bartolo

**Affiliations:** ^1^Ligue Nationale Contre le Cancer – Equipe Labellisée LIGUE 2018, Lymphocyte Cell Biology Unit, INSERM-U1221, Department of Immunology, Institut Pasteur, Paris, France; ^2^Collège Doctoral, Sorbonne Université, Paris, France

**Keywords:** TCR, signaling molecules, actin, microtubules, molecular transport, HIV-1, immunological synapse, polarity regulators

## Abstract

Dynamic localization of receptors and signaling molecules at the plasma membrane and within intracellular vesicular compartments is crucial for T lymphocyte sensing environmental cues, triggering membrane receptors, recruiting signaling molecules, and fine-tuning of intracellular signals. The orchestrated action of actin and microtubule cytoskeleton and intracellular vesicle traffic plays a key role in all these events that together ensure important steps in T cell physiology. These include extravasation and migration through lymphoid and peripheral tissues, T cell interactions with antigen-presenting cells, T cell receptor (TCR) triggering by cognate antigen–major histocompatibility complex (MHC) complexes, immunological synapse formation, cell activation, and effector functions. Cytoskeletal and vesicle traffic dynamics and their interplay are coordinated by a variety of regulatory molecules. Among them, polarity regulators and membrane–cytoskeleton linkers are master controllers of this interplay. Here, we review the various ways the T cell plasma membrane, receptors, and their signaling machinery interplay with the actin and microtubule cytoskeleton and with intracellular vesicular compartments. We highlight the importance of this fine-tuned crosstalk in three key stages of T cell biology involving cell polarization: T cell migration in response to chemokines, immunological synapse formation in response to antigen cues, and effector functions. Finally, we discuss two examples of perturbation of this interplay in pathological settings, such as HIV-1 infection and mutation of the polarity regulator and tumor suppressor adenomatous polyposis coli (Apc) that leads to familial polyposis and colorectal cancer.

## Introduction

Dynamic compartmentation of receptors and signaling molecules is key for T cells to sense environmental cues, trigger membrane receptors, and transduce and fine-tune intracellular signals controlling T cell migration, activation, and effector functions. This molecular compartmentation is ensured by the interplay between the plasma membrane, cytoskeleton networks, and intracellular organelles.

At the plasma membrane, dynamic assemblies of lipids and proteins form nano- to micro-scale domains that may become platforms for receptor signaling (i.e., cholesterol- and sphingolipid-enriched membrane domains or lipid rafts). These domains may facilitate either segregation or interaction between receptors (e.g., chemokine receptors and T cell receptors [TCRs]) and signaling molecules, conditioning their state of activation and preventing or facilitating receptor triggering and signaling (Lillemeier et al., 2006; Viola and Gupta, 2007; Simons and Gerl, 2010; Swamy et al., 2016). In addition, specific membrane phosphoinositides, transiently generated by enzymatic activation during chemokine receptor or TCR signaling, form different domains that target signaling effectors (e.g., Pleckstrin homology (PH) domain-containing proteins) at sites of receptor stimulation (Courtney et al., 2018).

The cortical actin cytoskeleton contributes to plasma membrane organization by generating areas of differential mobility of lipids and proteins. Thus, membrane-associated cytoskeletal fences shape the lateral distribution of membrane components involved in cell adhesion or receptor activation (Sako and Kusumi, 1995), adding a level of membrane organization cooperative with lipid microdomain partitioning. Furthermore, actin dynamics contribute to cell reorganization in response to chemokine or antigen stimulation needed for T cell migration, activation, and effector functions (Viola and Gupta, 2007; Nicolson, 2014; Niedergang et al., 2016). Although cortical actin and plasma membrane domains are often considered two-dimensional entities, three-dimensional membrane-cytoskeletal structures, such as microvilli, may form sensing exploratory extensions displaying receptor signaling components and adhesion molecules located within flexible subcellular areas distant from the cell body (Singer et al., 2001; Cai et al., 2017; Ghosh et al., 2020).

Several cellular organelles, including the Golgi apparatus and the endosomal and lysosomal compartments, continuously exchange with the plasma membrane. They contribute to lipid and protein sorting to subcellular areas involved in cell migration, activation, or secretion (Bretscher and Aguado-Velasco, 1998; Griffiths et al., 2010; Niedergang et al., 2016). Moreover, the endoplasmic reticulum (ER) and mitochondria contribute not only to protein synthesis and metabolism but also to T cell signaling (Quintana and Hoth, 2012).

Microtubules are crucial for intracellular transport and subcellular localization of molecules, vesicles, and organelles. They form a network that interacts with the nucleus, the cortical actin cytoskeleton, the plasma membrane, and various organelles, including endo-lysosomal compartments, the ER, and the Golgi apparatus. Microtubules coordinate the localization of proteins and organelles by means of their associated molecular motors, dynein, and kinesins. In this way, they ensure the dynamic relocalization of a variety of cellular components during T cell migration, activation, and effector functions (Vicente-Manzanares and Sanchez-Madrid, 2004; Niedergang et al., 2016; Martin-Cofreces and Sanchez-Madrid, 2018).

Intermediate filaments are the third major element of the cytoskeleton displaying different stabilities and mechanical properties from actin and microtubules. They cooperate with actin and microtubules in cellular architecture being important for cell polarization during migration, nuclear positioning, cellular mechanics, and cell adhesion-mediated mechano-transduction in various cell types (Etienne-Manneville, 2018). Their role in T cell biology remains poorly explored. In circulating T cells, vimentin intermediate filaments display a spherical pattern that relocalizes to a juxtanuclear area in chemokine-induced polarized cells. T cell rigidity (Brown et al., 2001), lymphocyte adhesion, transendothelial migration, and homing depend on intact intermediate filaments (Nieminen et al., 2006). In regulatory T cells (Tregs), vimentin intermediate filaments contribute to PKCθ localization at the distal pole of TCR-stimulated cells and to the control of Treg activity (McDonald-Hyman et al., 2018). In addition, vimentin regulates apoptosis in T cells during inflammation (Su et al., 2019). Septins are an additional component of the cytoskeleton in eukaryotic cells. These GTP-binding proteins assemble into hetero-oligomers that further associate forming higher order structures (e.g., filaments, bundles, and circles; Mostowy and Cossart, 2012). Recently, they have been shown to regulate several aspects of T cell biology, including signaling, differentiation, and cell division (Lassen et al., 2013; Sharma et al., 2013; Mujal et al., 2016). In particular, their role in regulating amoeboid T cell motility has been recently characterized (Tooley et al., 2009). Septins have been shown to regulate cortical rigidity and membrane dynamics. Their knockdown in T cells results in membrane blebbing and abnormal structure of both the leading edge and the uropod. These defects make T cell motility uncoordinated and poorly persistent (Tooley et al., 2009). The interplay of intermediate filaments and septins with actin and microtubules in T cells is not well defined, and it will not be further discussed in this review.

**FIGURE 1:**
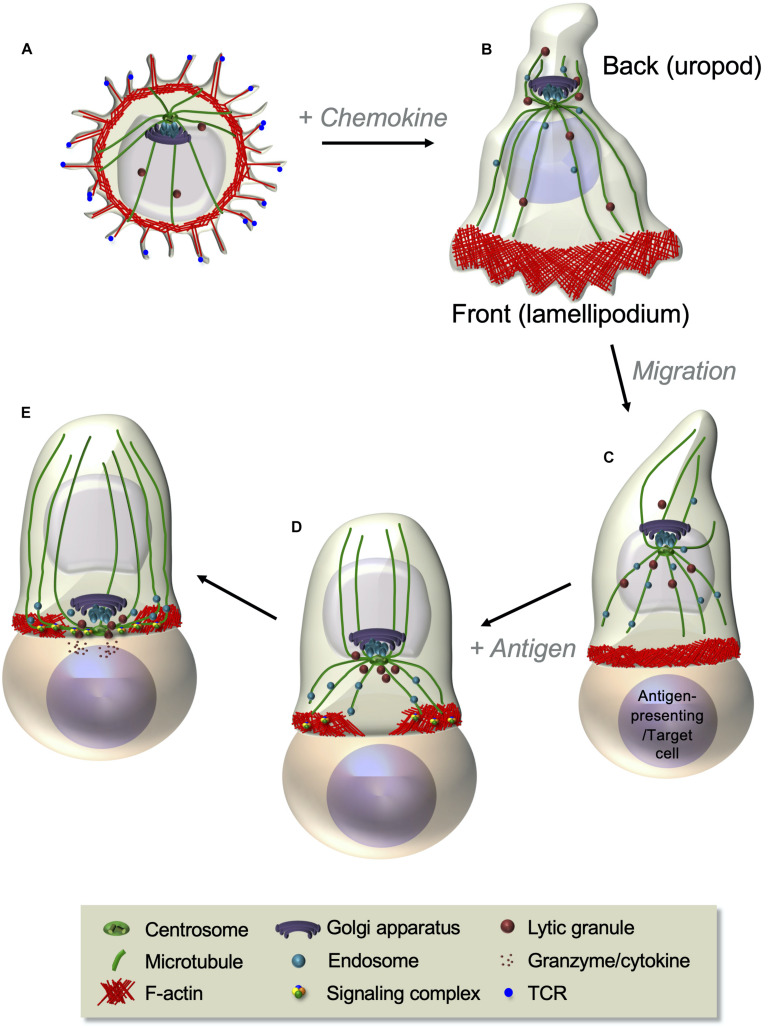
T cell polarization during T cell migration and immunological synapse formation. **(A,B)** Resting T cells, displaying microvilli at their surface **(A)**, polarize and start migrating in response to chemokines. A lamellipodium in which robust actin cytoskeleton dynamics takes place appears at the front edge, whereas a protrusion, named the uropod, forms at the back **(B)**. The centrosome is positioned between the nucleus and the uropod (see also Figure 3). Following chemokine gradients, T cells migrate through lymphoid organs or peripheral tissues where they meet antigen-presenting cells or target cells expressing their cognate antigen in complex with MHC proteins. **(C–E)** Upon antigen recognition, TCR signaling induces the coordinated polarization of actin and microtubule cytoskeletons. This is characterized by strong actin polymerization at the cell–cell contact site and the reorganization of the microtubule network that moves the centrosome toward the contact site **(C)**. Centrosome-associated organelles, such as the Golgi apparatus, endosomes, or lytic granules, move together with microtubules toward the contact site. Actin reorganizes while the T cell spreads at the contact site, forming a peripheral F-actin-enriched ring and a central F-actin poor area, where the centrosome and microtubule-associated organelles approach the cell–cell interface **(D)**. A final cytoskeleton-coordinated reorganization of the contact area generates the immunological synapse, where a concentration and dynamic clustering of TCRs, signaling and adhesion molecules, and co-signaling receptors occurs, thus ensuring sustained and controlled TCR signaling (further developed in Figure 4). In effector T cells, this is an area where cytokines or lytic granules are secreted **(E)**.

Among the most striking features of T cells is their capacity to rapidly change shape and profoundly reorganize their cellular interior leading to differential cell polarization in response to chemokine or antigenic stimuli (Figure 1). These cues induce coordinated changes in actin and microtubule cytoskeletons, membrane receptors, adhesion molecules, and various organelles that prepare the T cell to migrate in response to chemokines or to generate a signaling platform, the immunological synapse, in response to antigenic stimulation. Chemokine stimulation makes T cells to adopt a bipolar organization, with a lamellipodium at the migration front and a protruding uropod in the back differing in shape, cytoskeleton, and membrane component organization (Figures 1A,B). Such a remodeling prepares T cells to adhere and migrate through lymphoid organs and inflamed peripheral tissues (del Pozo et al., 1996). In turn, the encounter of T cells with antigen-presenting cells displaying cognate peptide antigen–major histocompatibility complex (MHC) complexes at their surface stabilizes the interaction between the two cells and triggers the formation of a highly organized and dynamic cell–cell interface named the immunological synapse (Figure 1C). Actin and microtubule cytoskeletons reorganize at the immunological synapse, together with TCR, co-stimulatory receptors, signaling molecules, and adhesion receptors. In addition, several organelles, such as the Golgi apparatus, the endosomal compartment, and the mitochondria, polarize to the immunological synapse releasing their cargo or retrieving membrane receptors and signaling molecules (Figures 1D,E). Altogether, the reorganization of molecular components at the immunological synapse ensures the control of T cell activation and effector functions (Niedergang et al., 2016).

The tight interplay between receptors and their signaling machineries, the actin and microtubule cytoskeleton, and intracellular molecular transport enables T cells to perform their functions, namely, sense environmental cues, polarize, migrate and patrol through lymphoid organs, recognize cognate antigen, and get activated to accomplish clonal expansion and differentiation into helper, regulatory, or cytotoxic T cells. Finally, it allows T cell effector functions, such as polarized secretion of cytokines to help B cells, and cytotoxic granules to eliminate infected or transformed cells. Various pivotal proteins facilitate the interplay between membrane, cytoskeletal, and organelle components. Among them, membrane–cytoskeleton linkers, such as the ezrin–radixin–moesin (ERM) family of proteins, talin, and several polarity regulators, play important roles at the different stages of T cell migration and immunological synapse formation (Krummel and Macara, 2006; Lasserre and Alcover, 2010; Garcia-Ortiz and Serrador, 2020).

**FIGURE 2:**
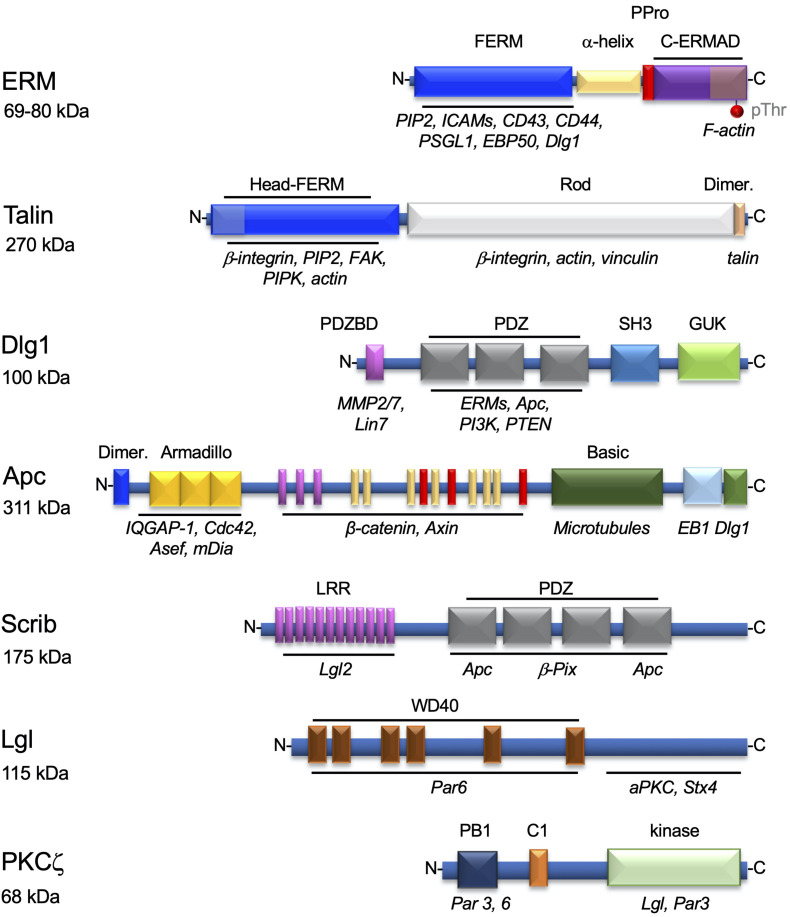
Proteins involved in the interplay between the plasma membrane components and the cytoskeleton in T cells. Structural organization of proteins regulating the interplay between membrane components and the actin and microtubule cytoskeletons. The modular domains involved in their interactions with lipids or other proteins are highlighted. Each domain, named on top, is shown in a different color and its interacting molecules depicted below in italics. ERMs and talin are mostly involved in the localization of adhesion proteins to particular areas of the plasma membrane, as the uropod (ERMs), or the immunological synapse periphery (talin). Dlg1, Apc, Scrib, Lgl, and PKCζ are polarity regulators involved in T cell migration and/or immunological synapse formation. For ERM, the phosphorylatable regulatory threonine residue (pThr) in the C-terminal domain is also shown. Molecular weights in kDa are show below each protein name.

Ezrin–radixin–moesin proteins bind plasma membrane components, such as phosphatidylinositol (4,5)-bisphosphate (PIP_2_) and transmembrane proteins, *via* their N-terminal FERM domain, and the cortical actin cytoskeleton *via* its threonine-phosphorylated C-terminal domain (Figure 2). Thus, ERMs help localizing membrane proteins at particular subcellular areas in various cell types (Arpin et al., 2011). T cells express ezrin and moesin that are important for confining TCRs and some of its signaling proteins to microvilli (Jung et al., 2016; Ghosh et al., 2020) and several adhesion proteins (i.e., intercellular adhesion molecules [ICAMs] and P-selectin glycoprotein ligand [PSGL]) to the uropod of migrating cells (Serrador et al., 1997, 1998, 2002). They can also link cortical actin with membrane rafts (Itoh et al., 2002). Finally, ezrin and moesin are key for immunological synapse formation and function (Allenspach et al., 2001; Delon et al., 2001; Roumier et al., 2001; Itoh et al., 2002; Faure et al., 2004; Shaffer et al., 2009; Lasserre et al., 2010). Other proteins also ensure the interplay between the plasma membrane and the actin cytoskeleton. For instance, talin and vinculin anchor adhesion proteins of the integrin family to the cortical actin cytoskeleton in areas of the cell in contact with integrin ligands in migrating cells and at the periphery of the immunological synapse (Jankowska et al., 2018; Figure 2).

Polarity regulators are multifunctional proteins displaying a variety of protein–protein interaction domains. These domains (e.g., PDZ domains) ensure interactions between polarity regulators themselves and with cytoskeleton components, cytoskeleton regulators (e.g., Cdc42), and membrane–cytoskeleton linkers, such as ERMs (Figure 2). Polarity regulators act in complexes. Several of them, such as Scribble, Dlg1, Lgl, PKCζ, Crumbs, PAR, and adenomatous polyposis coli (Apc), have been shown to control T cell polarization during migration, immunological synapse formation, or activation (Xavier et al., 2004; Ludford-Menting et al., 2005; Krummel and Macara, 2006; Real et al., 2007; Round et al., 2007; Bertrand et al., 2010; Lasserre et al., 2010; Aguera-Gonzalez et al., 2017).

In this review, we summarize the available knowledge on how the interplay between membrane receptor dynamics and signaling, the cytoskeleton, and intracellular vesicular compartments modulates three main aspects of T cell biology: T cell migration, immunological synapse formation in response to antigen stimulation, and effector functions. Finally, we describe two examples of perturbation of this interplay in pathological settings, i.e., HIV-1 infection and mutation of the polarity regulator and tumor suppressor Apc in familial polyposis and colorectal cancer.

## Cytoskeleton Interplay in Regulating T Cell Polarization and Migration

T cells are activated in lymph nodes, where they acquire the expression of specific tissue-homing receptors, such as adhesion and chemokine receptors, that sense information from the environment and lead T cell trafficking. Driven by the presence or the absence of these signals, T cells leave central lymphoid organs and undergo bloodstream navigation reaching peripheral lymph nodes or inflamed tissues. Their spherical shape facilitates the blood flux to push them forward. Moreover, the presence of thin protrusions on their surface, named microvilli, where some chemokine receptors, such as CXCR4, and adhesion molecules, such as L-selectins, are concentrated (Berlin et al., 1995; Singer et al., 2001) promotes sensing of the environment and the attachment necessary for them to slow down navigation and dock at a destination site. Once T cells have adhered to the blood vessel wall, chemokine stimulation induces the transient collapse of microvilli, and integrin activation leads to firm arrest, lymphocyte polarization, and transmigration through the vascular endothelial cell layer (Brown et al., 2003; Nijhara et al., 2004).

In the tissues, T cells modify their shape and adopt a different motility based on adhesion and on contact with the surrounding cells and the extracellular matrix. This allows them to migrate through tissues of different architecture and to interact with antigen-presenting cells (Moreau et al., 2018). This plasticity is fine-tuned by cytoskeleton structures, whose dynamics and interplay with molecular adaptors, such as cell polarity regulators, is essential for processes required for efficient T cell migration, including polarization, adhesion, and vesicle trafficking.

**FIGURE 3:**
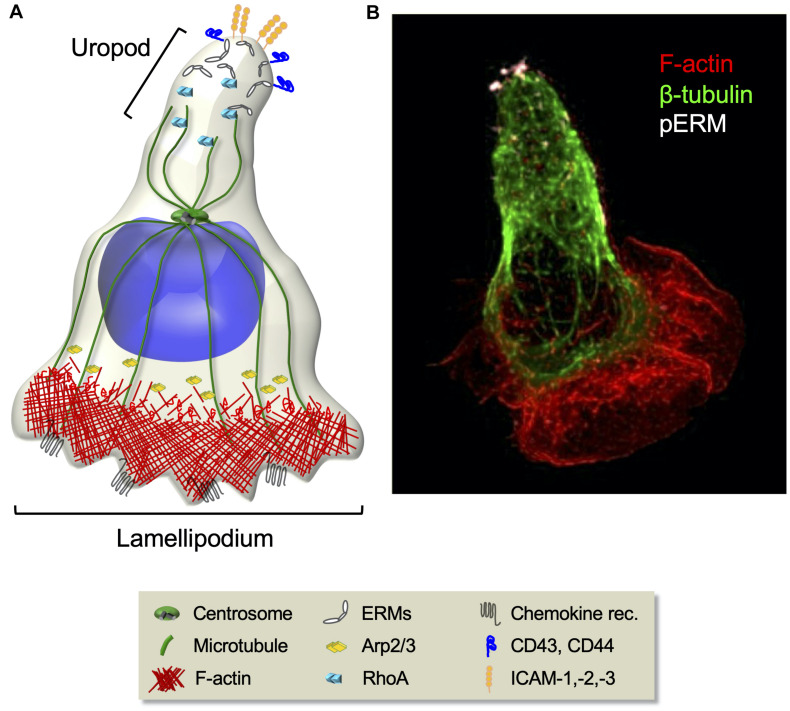
Cytoskeleton rearrangements during T cell migration. **(A)** Schematic representation of migrating T cell polarization involving the orchestrated rearrangement of both the actin and the microtubule cytoskeletons. At the cell front, chemokine stimulation induces the activation of the Arp2/3 effector complex that leads to actin polymerization and branching necessary for lamellipodium extension. At the rear, RhoA-dependent phosphorylation of ERM proteins induces their selective segregation to the uropod, where they recruit transmembrane adhesion molecules. **(B)** Fluorescence confocal microcopy image of a CEM T cell polarized in response to the chemokine SDF-1. F-actin (red), microtubules (green), and phosphorylated ERMs (white) are shown.

T cell polarization, an inherent requirement for migration, implies the formation of specialized subcellular areas, a lamellipodium at the leading edge and a uropod at the trailing edge (Figure 3). The leading edge, being enriched in chemokine receptors, guides the displacement, whereas the adhesive uropod supports cell–cell interactions. T cell migration relies on the mechanical cyclicity of lamellipodium extension and uropod retraction (Lauffenburger and Horwitz, 1996; Sánchez-Madrid and del Pozo, 1999).

### Cytoskeleton Rearrangements Shaping T Cell Polarization

The Rho family GTPases Cdc42, Rac1, and RhoA regulate actin and microtubules specialized dynamics at the front and the rear by transducing signals from surface receptors (Rougerie and Delon, 2012; Saoudi et al., 2014). At the cell front, chemokine stimulation induces the activation of Cdc42 and Rac1/2 *via* the phosphorylation of their guanine nucleotide exchange factors (GEFs), such as Vav1. These, in turn, engage several actin-binding proteins, trigger actin nucleation, and modulate the stability of filamentous actin (F-actin)-rich protrusions (reviewed in Dupre et al., 2015). In particular, Cdc42 and Rac1/2 induce the extension of filopodia and lamellipodia, respectively (Ridley et al., 2003; Dupre et al., 2015). Their function involves the activation of the WASP and WAVE proteins, followed by the activation of the Arp2/3 effector complex, that ensure actin polymerization and branching necessary for lamellipodium extension. Thus, defects in the Arp3 subunit are sufficient to affect the lamellipodium formation and the migratory behavior of CD8 T cells (Obeidy et al., 2020). In addition, the RhoA–ROCK pathway-dependent stimulation of actomyosin contraction is both responsible for the actin retrograde flow, on which lamellipodium extension and migration persistence rely on (Maiuri et al., 2015; Moreau et al., 2018), and essential for the detachment from the substrate (Alblas et al., 2001).

The precise control of microtubule organization in migrating lymphocytes is not fully understood, but their disassembly by nocodazole treatment disrupts cell polarity (Takesono et al., 2010). Events of microtubule growth and catastrophe may occur as described in other cell types (Hui and Upadhyaya, 2017). Interestingly, while migrating astrocytes or fibroblasts orient their centrosome between the nucleus and the front lamellipodium, migrating lymphocytes have their centrosome behind the nucleus (Ratner et al., 1997; Serrador et al., 1997; Lee et al., 2004; Figure 3). This positioning likely reflects some functional peculiarities of lymphocytes that need to be dissected.

In astrocytes and other non-leukocyte cell types, microtubule plus-end growth at the leading edge contributes to the lamellipodium activity by participating to the F-actin–membrane protrusion formation (Etienne-Manneville, 2004, 2013). Thus, microtubules drive vesicle exocytosis necessary for membrane extension (Bretscher, 1996), and their growth favors the increase in Rac1–GTP amounts, promoting the Rac1 signaling cascade (Liao et al., 1995; Waterman-Storer et al., 1999). In turn, Rac1/PAK1 activation may promote microtubule growth by inhibiting the microtubule-destabilizing protein Op18/stathmin (Wittmann et al., 2004). These features have only been partly described in T cells or leukocytes.

Microtubules contribute to RhoA activation at the rear of T cells. This involves the RhoGEF H1, which is sequestered by microtubules (Meiri et al., 2012), and the subsequent activation of the RhoA–ROCK pathway and phosphorylation of myosin light chain, which induces uropod contraction (Chang et al., 2008; Kaverina and Straube, 2011; Yoo et al., 2012). The RhoA–ROCK pathway also contributes to activate the formin mDia, an actin nucleator that regulates peripheral actin flow (Otomo et al., 2005). Hence, microtubule dynamics in the front contributes to the persistence of the actin flow (Park and Doh, 2015), whereas microtubule stability at the rear is required for myosin light chain-dependent uropod contraction, providing the mechanical force necessary for effective cell locomotion.

Ezrin–radixin–moesin proteins, which ensure interactions between cortical actin and membrane components, are key for chemokine-induced T cell polarization. Chemokines induce transient ERMs de-phosphorylation, dissociation from the plasma membrane and the actin cytoskeleton, and release of GEF proteins that in turn activate Rac1 and Cdc42. This supports F-actin polymerization at the protrusive leading edge (Hao et al., 2009; Garcia-Ortiz and Serrador, 2020). Then, RhoA-dependent re-phosphorylation of ERMs induces their selective segregation to the uropod, where they recruit adhesion molecules, such as ICAM-1, -2, -3, CD44, and PSGL-1 (Figure 3). Ezrin and moesin FERM domains interact with a consensus sequence in the intracellular region of these adhesion molecules. Phosphorylated ERMs constitute a functional polar cap in the rear pole *via* their cooperation with lipid raft-associated flotillins (Lee et al., 2004; Martinelli et al., 2013) where they re-activate RhoA and myosin, modulating contractility at the uropod in a positive feedback loop.

### Membrane–Cytoskeleton Interactions During T Cell Adhesion and Migration

Cell membrane components participate to adhesion and migration, acting as sensors of the environment and converting external signals into biochemical messages for the cell. Lipid rafts of different composition redistribute during T cell polarization in response to chemokines, being enriched in the ganglioside GM3 at the leading edge and in GM1 in the uropod (Gomez-Mouton et al., 2001). This contributes to the spatial segregation of chemokine receptors or adhesion molecules and to their interaction with cytoskeleton structures and/or signaling complexes, thus influencing their spatiotemporal activation (reviewed in Dustin et al., 2004; Manes and Viola, 2006). While front GM3-enriched rafts mainly concentrate chemokine receptors, such as CXCR4 and CCR5, GM1-enriched rafts colocalize with the adhesion protein CD44 at the uropod, where ERM-associated flotillins are found as well (Gomez-Mouton et al., 2001). Integrin activation depends on their localization in ganglioside GM1-containing rafts (Gomez-Mouton et al., 2001). Integrins are present not only in the uropod of polarized T cells but also in a larger zone in contact with their ligands (Gomez-Mouton et al., 2001; Leitinger and Hogg, 2002; Smith et al., 2005). Indeed, integrins move laterally within lipid rafts, and their activation state may result in the localization in different cell compartments, including the leading edge (Hogg et al., 2003; Hyun et al., 2009).

Integrins represent the main class of adhesion molecules responsible for interactions with both the extracellular matrix and neighboring cells. They are heterodimeric proteins whose activation relies on their reversible conformational changes triggered by surface receptors, including the TCR and chemokine receptors, or by their own binding to multivalent ligands (reviewed by Baker and Koretzky, 2008; Abram and Lowell, 2009). In addition, both lipid raft microenvironment and cytoskeleton interactions shape integrin activation by controlling single hotspots of integrins in the membrane and their clustering in larger plasma membrane domains (Stewart et al., 1998; Leitinger and Hogg, 2002; Cairo et al., 2006; van Zanten et al., 2009). Integrin clustering selectively provides higher avidity for ligands (Stewart and Hogg, 1996; van Kooyk et al., 1999), although it does not change their affinity (Kim et al., 2004; Luo et al., 2005). It results from the TCR-mediated signaling (Abram and Lowell, 2009) and may be negatively regulated by GTPases. Indeed, inhibition of the RhoA–ROCK pathway induces clustering of lymphocyte function-associated antigen-1 (LFA-1), followed by the induction of adhesion to its ligand, ICAM-1 (Rodriguez-Fernandez et al., 2001).

Integrin activation state in turn influences the composition of the surrounding environment, thus impacting the downstream signaling and enabling cytoskeleton remodeling (Schwartz, 2010; Byron et al., 2015). Whereas active β1 integrins are mainly found in complexes with actin and microtubule-associated proteins, such as talin and kindlin, inactive integrins form complexes with molecules involved in adhesion and cytoskeleton organization (Rho and Ras GTPase family members) or in membrane trafficking (Arf and Rab GTPases) in K562 leukemic cells, which may resemble to T cells for their adhesion pattern (Byron et al., 2015).

Interestingly, the link between integrins and the cytoskeleton is bidirectional, and their functions are reciprocally modulated (Vicente-Manzanares et al., 2009). For instance, LFA-1 activation during cell migration is modulated by physical forces on its β subunit applied by the actin cytoskeleton (Nordenfelt et al., 2016). Moreover, the inhibition of actin polymerization by cytochalasin D prevents the formation of new nascent adhesions (Choi et al., 2008), whereas microtubule regrowth after nocodazole washout correlates with adhesive structure disassembly (Kaverina et al., 1998; Ezratty et al., 2005).

T cell adhesion to the substrate and subsequent changes on the physical properties of their membranes are also sensed by BAR domain-containing proteins that translate these signals into cytoskeleton remodeling. Substrate attachment of the adhesive uropod of neutrophils induces a membrane curvature critical for the activation of the SRGAP2 BAR protein, in keeping with the notion of phospho-ERMs asymmetrical segregation at the uropod during T cell migration (Ren et al., 2019). Hence, the rear membrane curvature would be responsible for the activation of specific BAR proteins and then kinases, determining the local phosphorylation of ERMs and their membrane binding at the rear (Ren et al., 2019). It is noteworthy that the BAR protein CIP4, involved in membrane deformation during endocytosis, is also crucial for integrin-dependent activation of WASP. Indeed, T cells from CIP4^–/–^ mice present defects in adhesive interactions, impairing transmigration across endothelial cell monolayers (Koduru et al., 2010).

### Intracellular Traffic in T Cell Adhesion and Migration

Intracellular trafficking may promote the polarization of motile lymphocytes by allowing the dynamic turnover of membrane and the delivery of cargos, such as chemokine or cytokine receptors and integrins, to specific subcellular localizations. Cargos are transported along actin and microtubule structures *via* myosin, kinesin, and dynein molecular motors, respectively, and may be associated with vesicles. Integrins continuously cycle between the plasma membrane and endosomal compartments (Paul et al., 2015). Clustering of integrins in lipid rafts may contribute to their internalization and recycling, possibly facilitating integrin targeting at the leading edge (Hyun et al., 2009) where they would establish adhesion during migration. These processes are poorly elucidated in T cells, and most of the information is on LFA-1. In the uropod of T cells migrating on ICAM-1, LFA-1 undergoes a caveolar endocytosis, which is regulated by G-protein-coupled receptor, mediated for instance by Gαq/11 (Svensson et al., 2012). Partitioning into lipid rafts is likely pivotal for LFA-1 to undergo a caveolae-dependent endocytic pathway (Upla et al., 2004; Fabbri et al., 2005). Moreover, inhibition of the small GTPases Rab13, a key regulator of intracellular membrane trafficking, could reduce LFA-1-dependent adhesion on ICAM-1 and the formation of micro-adhesion rings of LFA-1 at the contact site with antigen-presenting cells (Nishikimi et al., 2014), essential for T cell activation (Hashimoto-Tane et al., 2016) (see section “Actin–Microtubule Interplay Shaping T Cell Effector Functions”).

## T Cell Sensing of Antigen Cues, TCR Triggering, and Immunological Synapse Formation

### Topological Distribution of the TCR

Once in the lymph nodes or in peripheral tissues, T cells scan antigen-presenting cells searching for cognate peptide–MHC complexes. The localization of the TCR and some of its proximal signaling molecules on microvilli may enhance the sensing capacity of T cells.

Mapping TCRs localization relative to the 3D membrane topology demonstrated that TCRs are segregated on the tips of microvilli in fixed resting and effector T cells (Jung et al., 2016). CD3ε follows the same distribution than TCR, and both proteins significantly colocalize with L-selectin, further confirming their localization at microvilli tips. This approach has been recently extended to analyze the distribution of additional membrane receptors and signaling proteins in human effector T cells and the Jurkat T cell line (Ghosh et al., 2020). It has been shown that the majority of the CD3ζ subunit, the co-receptor CD4, and the adhesion protein CD2 are localized to microvilli. The protein kinase Lck and the adaptor LAT are also enriched in microvilli, although a significant fraction of these molecules is found outside these structures. This observation agrees with Lck and LAT being partially associated with intracellular vesicular compartments (Soares et al., 2013; see also “Alterations of T Cell Cytoskeleton and Molecular Traffic in Pathological Settings” section). On the contrary, the protein tyrosine phosphatase CD45, which inhibits TCR/CD3 complex phosphorylation, is segregated from the TCR, hence mostly excluded from microvilli (Razvag et al., 2018; Ghosh et al., 2020).

The structural integrity of microvilli requires an intact actin cytoskeleton, and the confinement of proteins into microvilli is dependent on membrane–cytoskeleton linker proteins of the ERM family (Ghosh et al., 2020). Indeed, phosphorylated ERMs are concentrated into microvilli where they co-localize with F-actin and TCRs. Additionally, overexpression of a dominant-negative form of the ERM member ezrin in Jurkat T cells results in the disappearance of membrane protrusions and redistributions of microvilli-associated proteins throughout plasma membrane. These modifications correlate with a reduction of TCR-dependent signaling, as measured by the inhibition of the phosphorylation of ERK kinases (Ghosh et al., 2020).

Studies of microvillar dynamics in live T cells indicated that most microvilli undulate and move laterally, allowing a faster and more efficient scanning of the antigen-presenting cell surface (Cai et al., 2017). Microvillar dynamics is slowed down once the contact with the antigen-presenting cells is stabilized, likely as a consequence of TCR engagement by peptide–MHC complexes and integrin activation. Further analyses demonstrated that signaling complexes containing the TCR and the ZAP70 protein kinase colocalized in areas corresponding to microvillar tips (Cai et al., 2017), suggesting that the geometry and dynamics of signaling protein complexes or pre-existing “protein islands” described before (Lillemeier et al., 2010) are actually influenced by membrane 3D topology.

Collectively, these data indicate that concentration of TCRs, associated co-receptors, and signaling proteins at microvilli tips plays a critical role in antigen recognition and early activation steps. Indeed, this organization and the mobility of membrane protrusions would allow a “topological scan” of antigen-presenting cell surface, increasing speed and efficiency of antigen search (Cai et al., 2017). Moreover, focusing TCR and its signaling machinery to microvilli increase the avidity of interaction of antigen receptors with peptide–MHC complexes and facilitate early signal transduction. However, at later steps of activation, ERMs dephosphorylation may lead to microvilli resorption (Ghosh et al., 2020), thus favoring mixing of signaling proteins with TCRs and centripetal movement of signaling complexes, followed by their internalization and/or dissociation.

It is worth noting that microvilli might also have additional functions, such as the recently described generation of extracellular organelles or “immunological synaptosomes,” through a mechanism similar to trogocytosis (Kim et al., 2018). These entities may carry various signals to the antigen-presenting cells (e.g., TCR/CD3 complexes, co-stimulatory proteins, and cytokines) and are probably related to the extracellular vesicles previously detected at the center of the immunological synapse (Choudhuri et al., 2014).

### Immunological Synapse Formation

The early consequence of a productive TCR engagement by its cognate antigen displayed on the surface of an antigen-presenting cell is twofold. First, the T cell stops or slows down its movement, and then it starts polarizing toward the antigen-presenting cell. These initial events, driven by rearrangements of the actin and microtubule cytoskeletons, result in the formation of the immunological synapse. This specialized interface allows the communication between the two cells involved, ensuring efficient TCR signal transduction leading to T cell activation, clonal expansion, and differentiation.

**FIGURE 4:**
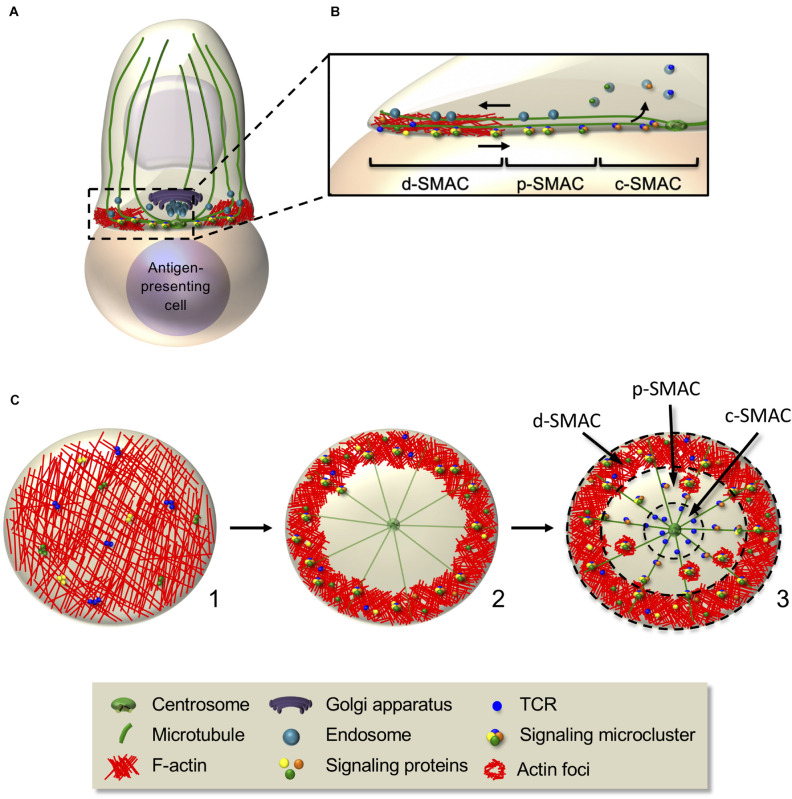
Interplay between actin and microtubule cytoskeleton controls signaling microcluster dynamics at the immunological synapse. **(A,B)** Schematic representation of cellular and molecular rearrangements leading to immunological synapse formation. This involves actin and microtubule rearrangements and organelle polarization, driving to the generation of dynamic signaling microclusters. The latter form in the d-SMAC, at the periphery of the immunological synapse **(B)**, then centripetally move to the center in an actin- and microtubule-dependent manner. Some molecules (e.g., the TCR) coalesce in the center generating the c-SMAC, whereas others are internalized or disassembled in the p-SMAC during their centripetal movement. **(C)** *En-face* view of an immunological synapse showing the kinetics of its reorganization. F-actin, which is initially disseminated throughout the synapse (1), redistributes and concentrates in the peripheral area, whereas microtubules adopt a radial organization from the centrosome to the periphery (2). Signaling microclusters form at the synapse periphery and move centripetally (2,3), first by retrograde actin waves, then by the microtubule-based motors dynein. Adhesion rings (not shown) and F-actin foci transiently surround signaling microclusters, reminiscent of “micro-synapses” (3). Dashed circles in (3) separate d-SMAC, p-SMAC, and c-SMAC.

The immunological synapse is characterized by intensive F-actin polymerization at the interface with the antigen-presenting cell. Once the synapse is stabilized, F-actin clears from the center of the synapse leaving an actin-rich peripheral ring (Bunnell et al., 2001; Ritter et al., 2015). Microtubules also reorganize at the immunological synapse, some irradiating from the centrosome and oriented toward the periphery of the synapse where some appear to anchor and bend (Kuhn and Poenie, 2002; Lasserre et al., 2010; Aguera-Gonzalez et al., 2017). Concomitantly, the centrosome translocates toward the center of the synapse, beneath the plasma membrane, within a minute after F-actin clearance (Geiger et al., 1982; Kupfer et al., 1986; Stinchcombe et al., 2006; Ueda et al., 2011; Ritter et al., 2015; Figure 4). The exact molecular mechanism moving the centrosome to the synapse is still not clear. Interaction of microtubules with the actin cortex at the synapse periphery *via* ezrin and Dlg1 appears to facilitate centrosome polarization (Lasserre et al., 2010). A process of microtubule bending at the synapse periphery mediated by the motor dynein has been proposed to facilitate microtubule tension and centrosome docking close at the synapse center (Kuhn and Poenie, 2002). Decreased F-actin polymerization at the centrosome could also allow its detachment from the nucleus and its translocation, as shown in B cells (Obino et al., 2016). In turn, microtubules and the centrosome could control the F-actin remodeling at the synapse, as centriole depletion impairs actin clearance (Tamzalit et al., 2020). By converging toward the centrosome at the center of the synapse, microtubules guide polarized transport of vesicular components and organelles, such as the Golgi and several endosomes and secretory lysosomes (Kupfer and Dennert, 1984; Das et al., 2004; Chemin et al., 2012). The clearance of F-actin at the center of the synapse may be related to the localization of this secretory machinery. For instance, nitric oxide synthase-mediated post-translational modifications of actin may remodel the actin cytoskeleton by controlling polymerization/depolymerization (Garcia-Ortiz et al., 2017).

T cells can form simultaneously multiple synapses, integrating signal from several antigen-presenting cells, but polarize their cytokine secretory machinery mainly toward the one displaying the strongest stimulus (Depoil et al., 2005). In some instances, the T cell does not completely stop and forms asymmetric and not stabilized synapses, while it continues to move over the antigen-presenting cell. In this case, the cell–cell contact zone is called immunological kinapse. As in migrating cells, T cell presents an F-actin-rich lamellipodium, and the centrosome and vesicular components are localized at the uropod (reviewed by Fooksman et al., 2010). Interestingly, kinapses still permit durable interactions and TCR signal integration (Skokos et al., 2007; Moreau et al., 2012; Mayya et al., 2018). T cells may cycle between synapse and kinapse *in vitro* and *in vivo*, depending on the stimulation level, which may facilitate T cell interaction with several antigen-presenting cells (Sims et al., 2007; Moreau et al., 2015).

### TCR Signaling Drives Cytoskeleton Reorganization

The initial TCR signaling occurring during the immunological synapse formation proceeds through serial reactions to control cytoskeleton reorganization. TCR-associated CD3 subunits are phosphorylated in their cytoplasmic regions on tyrosine-containing signaling motifs named immunoreceptor tyrosine-based activation motifs (ITAMs) (Barber et al., 1989; Reth, 1989). ITAM phosphorylation by Lck, a membrane-associated protein kinase of the Src family, induces ZAP70 recruitment to CD3 and its activation (Iwashima et al., 1994). Then, ZAP70 phosphorylates LAT, which in turn recruits SLP76 (Finco et al., 1998; Yablonski et al., 1998b; Zhang et al., 1998). Centrosome and microtubule repositioning requires efficient recruitment and activation of all these proteins (Lowin-Kropf et al., 1998; Kuhné et al., 2003; Tsun et al., 2011). Phosphorylated SLP76 binds the GEF Vav and the adaptor protein Nck (Wu et al., 1996; Wunderlich et al., 1999). The second signal received by T cells through the co-stimulatory molecule CD28 also allows the recruitment of Nck and Vav that bind to CD28 and can be activated in a TCR-independent manner upon CD28 engagement (Acuto et al., 2008). Vav activates the Rho family GTPases Rac1 and Cdc42 that together with Nck recruit and activate WAVE2 and WASP. As in migrating cells, WAVE2 and WASP then stimulate Arp2/3 ensuring actin polymerization and branching (Blumenthal and Burkhardt, 2020). Interestingly, WASP, together with PKCθ, controls the conversion of kinapses into synapses, as WASP^–/–^ T cells cannot reform symmetric stable interaction with stimulatory surfaces after a cycle of migration (Sims et al., 2007). Vav, Rac1, Arp2/3, and formins have been involved in centrosome translocation, likely regulating the interplay between actin and microtubule networks (Ardouin et al., 2003; Gomez et al., 2007; Randzavola et al., 2019). TCR-induced signaling recruits at the synapse and activates actin cytoskeleton regulators involved in its polarization, cortical reorganization, and maintenance, such as dynamin 2, the cortactin homologue HS1, and the polarity regulator Dlg1 (Gomez et al., 2005, 2006; Round et al., 2005). Finally, clathrin accumulation at the synapse recruits the actin-polymerization machinery, indicating a relationship between the endocytic machinery and actin dynamics (Calabia-Linares et al., 2011).

Initial TCR triggering modifies the membrane phospholipid composition that controls F-actin organization at the synapse. LAT recruits PLCγ1, which metabolizes PIP_2_, generating the second messengers diacyl glycerol (DAG) and inositol (1,4,5)-trisphosphate (IP_3_), that respectively activate PKCs and calcium release from intracellular stores. CD28 recruits the phosphoinositide-3-kinase (PI3K), which converts PIP_2_ into phosphatidylinositol (3,4,5)-trisphosphate (PIP_3_). Both PIP_2_ and PIP_3_ regulate F-actin localization at the immunological synapse. Indeed, F-actin depletion from the center of the synapse correlates with a reduction of PIP_2_ at the plasma membrane (Ritter et al., 2015; Gawden-Bone et al., 2018), whereas generation and maintenance of the actin-rich ring is controlled by the annular accumulation of PIP_3_ at the synapse periphery (Le Floc’h et al., 2013).

DAG plays an important role in centrosome polarization (Quann et al., 2009; Liu et al., 2013; Chauveau et al., 2014). The mechanisms involved in microtubule anchoring at the synapse periphery and centrosome reorientation are complex and regulated by various effectors, underscoring the interplay between actin and microtubule cytoskeleton. These include membrane–microfilament linkers, such as ezrin, molecular motors, such as dynein, and polarity regulators, such as Dlg1 and Apc (Combs et al., 2006; Stinchcombe et al., 2006; Gomez et al., 2007; Martin-Cofreces et al., 2008; Bertrand et al., 2010; Lasserre et al., 2010; Liu et al., 2013; Aguera-Gonzalez et al., 2017). However, the interplay between these effectors is poorly understood.

### Signaling Complexes Assembly and Regulation by the Cytoskeleton

Early TCR and co-stimulatory molecule signaling is responsible for bringing the actin polymerization machinery, regulators of its organization, and the centrosome and microtubules to the immunological synapse (Figure 4). However, a positive feedback loop exists since actin and microtubule cytoskeletons are in turn necessary for maintaining TCR signaling. They regulate the spatiotemporal organization of the signaling machinery, not only reinforcing and sustaining signaling but also driving TCR signal downregulation (Nguyen et al., 2008; Lasserre et al., 2010).

Initially, TCRs, signaling and adhesion molecules, as well as cytoskeleton structures, are not uniformly distributed at the plasma membrane, possibly reflecting their distribution in microvilli (Jung et al., 2016; Cai et al., 2017; Ghosh et al., 2020). Then, they coalesce into concentric supramolecular activation clusters (SMACs) (Monks et al., 1998; Grakoui et al., 1999). A central-SMAC (c-SMAC) is enriched in TCR and associated proteins, such as CD3, co-signaling receptors, such as CD2 and CD28, inhibitory receptors, such as CTLA-4 and PD1, and their downstream signaling proteins (reviewed in Dustin and Choudhuri, 2016). Surrounding the c-SMAC, the peripheral SMAC (p-SMAC), containing integrins, such as LFA-1, and its cytoskeleton linkers as talin (Monks et al., 1998; Grakoui et al., 1999), stabilizes the synapse (Comrie et al., 2015). Finally, the distal SMAC (d-SMAC) contains large proteins, such as the protein tyrosine phosphatase CD45 (Davis and van der Merwe, 2006; Cordoba et al., 2013). The d-SMAC also corresponds to the peripheral actin ring and is enriched in microtubule linkers (e.g., IQGAP-1 and ezrin) (Roumier et al., 2001; Watanabe et al., 2004; Stinchcombe et al., 2006; Lasserre et al., 2010). This SMAC-type organization was mostly observed *in vitro* on stimulatory surfaces made of planar bilayers displaying ICAM-1 and MHC–peptide antigen molecules or using B cells as antigen-presenting cells. Indeed, when reducing the concentration of antigenic peptide or costimulatory molecules or studying different physiological conditions (e.g., T cells in different differentiation states and/or interacting with different antigen-presenting cells), the spatiotemporal pattern is highly diverse (reviewed in Thauland and Parker, 2010). For instance, in the case of the asymmetrical contacts formed in kinapses, the molecular organization at the uropod is reminiscent of the c-SMAC (reviewed in Dustin, 2008).

Upon initial TCR triggering, Lck, ZAP70, SLP76, and LAT are recruited at the plasma membrane close to the area of TCR stimulation (see “Alterations of T Cell Cytoskeleton and Molecular Traffic in Pathological Settings” section). Some of these molecules (e.g., TCR and LAT) are pre-clustered in separate stable domains before TCR stimulation that mix upon TCR engagement (Lillemeier et al., 2010; Beck-Garcia et al., 2015). Studying immunological synapse formation using activating planar bilayers as surrogate antigen-presenting cells and live cell TIRF microscopy revealed that once at the plasma membrane, these signaling molecules nucleate into dynamic microclusters in the d-SMAC where they are phosphorylated (Lee et al., 2002) and rapidly engage into a centripetal movement (Bunnell et al., 2002; Campi et al., 2005; Yokosuka et al., 2005; Varma et al., 2006; Kaizuka et al., 2007). The F-actin-rich ring acts as a scaffold for microcluster assembly and stabilization (Campi et al., 2005), whereas the microtubules seem to be dispensable for microcluster formation but needed for their centripetal movement (Lasserre et al., 2010; Hashimoto-Tane et al., 2011; Figures 4B,C).

Signaling microclusters have been shown to be surrounded by adhesion molecules similar to the p-SMAC and by F-actin enrichments, called foci (Kumari et al., 2015; Hashimoto-Tane et al., 2016; Figure 4C). The adhesion ring formation depends on LFA-1 signaling and actin dynamics, whereas actin foci are regulated by WASP (Kumari et al., 2015; Hashimoto-Tane et al., 2016). These observations suggest the existence of transient “micro-synapses” within the immunological synapse with similar structure but at a smaller scale. They likely provide scaffolds for TCR and signaling molecules clustering, promoting efficient signaling (Pageon et al., 2016).

Impairing actin cytoskeleton meshwork alters microcluster formation and TCR signaling. For instance, TCR and SLP76 microclusters do not form in T cell treated with latrunculin-A that depolymerizes F-actin (Campi et al., 2005; Babich et al., 2012). Furthermore, impairing F-actin dynamics, with jasplakinolide that stabilizes filaments, alters the centripetal movement of SLP76 microclusters, which cannot reach the c-SMAC (Babich et al., 2012). Accordingly, downstream events, such as calcium flux, NFAT1 activation, and interleukin (IL)-2 transcription, are also altered by actin inhibitors, although with differential effects depending on the dose used (Nolz et al., 2007). Similarly, to the events taking place in T cell migration, F-actin continuously pulls forces on the plasma membrane and the antigen-presenting cell due to contraction dependent on the molecular motor myosin II. Additionally, actin polymerization pushes forces and drives the retrograde flow of the actin network.

Microtubules have been recently involved in the regulation of these forces. Indeed, T cell treated with nocodazole displayed more sustained actin flow on activating planar bilayers (Hui and Upadhyaya, 2017). Together, these forces stabilize the actin cytoskeleton meshwork, allow the formation of the integrin-rich p-SMAC, and maintain the radial symmetry of the immunological synapse (Campi et al., 2005; Nguyen et al., 2008; Ilani et al., 2009; Hashimoto-Tane et al., 2011; Husson et al., 2011; Babich et al., 2012; Comrie et al., 2015). Mechanical forces and waves of actin polymerization also initiate the centripetal movement of signaling microclusters toward the p-SMAC (Campi et al., 2005; Yokosuka et al., 2005; Nguyen et al., 2008; Ilani et al., 2009; Yi et al., 2012; Comrie et al., 2015; Murugesan et al., 2016) and their segregation into the c-SMAC where signaling terminates (Lee et al., 2003; Varma et al., 2006; Kumari et al., 2012). Some signaling molecules (e.g., SLP76, LAT, and ZAP70) are downregulated in the p-SMAC, before reaching the c-SMAC (Yokosuka et al., 2005; Lasserre et al., 2011), whereas the TCR is downregulated in the c-SMAC by internalization (Lee et al., 2002, 2003; Varma et al., 2006; Vardhana et al., 2010) or by accumulation into extracellular vesicles (Choudhuri et al., 2014; Saliba et al., 2019).

Impairing the microtubule cytoskeleton alters microcluster centripetal movement (Bunnell et al., 2002; Lasserre et al., 2010; Hashimoto-Tane et al., 2011). For instance, SLP76 microclusters do not move to the c-SMAC in T cells silenced for ezrin or the polarity regulators Dlg1 and Apc that display altered microtubule network organization at the synapse (Lasserre et al., 2010; Aguera-Gonzalez et al., 2017). Likewise, perturbing the microtubule-associated molecular motor dynein impairs centripetal TCR microcluster movement (Hashimoto-Tane et al., 2011). In addition, knockdown of the microtubule end-binding protein 1 (EB1) alters TCR dynamics at the immunological synapse and downstream signaling (Martin-Cofreces et al., 2012).

Importantly, impairing or slowing down microcluster movement toward the center of the synapse correlates with enhanced T cell signaling (e.g., higher level of phosphorylated LAT at the synapse and higher activation of Erk1/2), indicating that microcluster dynamics is linked to TCR signal downregulation (Mossman et al., 2005; Nguyen et al., 2008; Lasserre et al., 2010; Hashimoto-Tane et al., 2011). The molecular mechanisms involved in signaling complex deactivation and their relationship with microcluster centripetal movement are not fully understood. Several mechanisms may coexist at the synapse, including tyrosine dephosphorylation in the c-SMAC by the presence of the CD45 phosphatase (Varma et al., 2006) or post-translational modifications of signaling complexes facilitating their disaggregation (Lasserre et al., 2011).

In conclusion, while dynamic F-actin first initiates the formation of signaling microclusters, it subsequently leads to signaling molecule deactivation by targeting them to the c-SMAC, in close cooperation with the microtubule network. Therefore, a fine-tuned interplay between both cytoskeletons is key for sustaining TCR signaling and for conditioning its intensity and duration.

### Vesicle Traffic Controls TCR Signaling and the Cytoskeleton

Targeting of organelles and intracellular vesicular compartments to the immunological synapse regulates T cell signaling and effector functions, as well as participates to the communication between T cells and antigen-presenting cells. Indeed, TCR–CD3 and two of its proximal signaling molecules, Lck and LAT, not only are localized at the plasma membrane, in part in microvilli, but also are present in endosomal and Golgi compartments. These molecules partition differently between plasma membrane and intracellular compartments, and their targeting to the immunological synapse is uniquely regulated. Targeting of vesicles carrying CD3ζ, Lck, and LAT to the immunological synapse follows TCR triggering and the formation of early microclusters containing phosphorylated forms of these proteins (Blanchard et al., 2002a; Ehrlich et al., 2002; Bonello et al., 2004; Balagopalan et al., 2013, 2018). This is consistent with the role of plasma membrane pools of these molecules in the initial signal triggering and of vesicular pools in signal amplification by fueling additional signaling molecules to the immunological synapse.

TCR–CD3 components exchange between the plasma membrane and recycling endosomes. Interestingly, although part of the same multi-subunit TCR–CD3 complex, the CD3ζ chain has a different turnover, and it is more concentrated in the endosomal compartment than in other subunits (reviewed in Alcover et al., 2018). Clustering of TCR–CD3 complexes at the synapse is maintained by microtubule-dependent polarized vesicle traffic (Blanchard et al., 2002a; Das et al., 2004; Soares et al., 2013) and controlled by several regulatory proteins. These include intraflagellar transport proteins and the microtubule-binding protein EB1, which interact with microtubules and TCR–CD3 components (Finetti et al., 2009; Martin-Cofreces et al., 2012), several Rab GTPases, and vesicle fusion regulators, such as the SNAREs VAMP-3, SNAP-23, syntaxin-4, and the calcium sensor synaptotagmin-7 (Das et al., 2004; Patino-Lopez et al., 2008; Finetti et al., 2009, 2015; Soares et al., 2013; Onnis et al., 2015). Altered expression of some of these regulators results in reduced TCR signaling and T cell activation (Finetti et al., 2009; Martin-Cofreces et al., 2012). Intraflagellar transport proteins are key for the formation of the primary cilium, a sensory structure present in many cell types. Although T cells lack primary cilia, they use the same molecular machinery, including IFT20, IFT57, and IFT88 proteins, to transport TCR–CD3 complexes to the synapse (Finetti et al., 2009). Likewise, T cells express and utilize SNARE proteins involved in vesicle fusion in other secretory cellular systems (Sudhof and Rizo, 2011) to control polarized traffic to the immunological synapse (Das et al., 2004; Soares et al., 2013; Finetti et al., 2015). Proteins controlling actin polymerization and branching, such as ARPC2 (Zhang et al., 2017) or WASH (Piotrowski et al., 2013), can also modulate TCR endosomal trafficking and its polarization, thus affecting T cell homeostasis and function.

Lck is partly associated with endosomes, and contrary to CD3ζ and LAT, its plasma membrane pool is bigger than the endosomal one (Soares et al., 2013). Intracellular Lck is mainly localized in the Rab11^+^ recycling endosomal compartment (Soares et al., 2013; Bouchet et al., 2017). It constitutively recycles between the plasma membrane and pericentrosomal endosomes, and it is targeted to the immunological synapse soon after TCR engagement *via* endosomal polarization (Ehrlich et al., 2002; Anton et al., 2008). The Rab11 effector FIP3 (Rab11 family interacting protein-3) controls Lck subcellular localization, its clustering at the immunological synapse, and its signaling functions. FIP3 links Rab11 with microtubule molecular motors, such as dynein and kinesin, and with components of the exocyst complex controlling endosomal traffic (Horgan and McCaffrey, 2009). Interestingly, FIP3-mediated Lck localization conditions both basal and TCR-mediated phosphorylation of Lck substrates and intracellular calcium (Bouchet et al., 2017). Moreover, perturbing Lck endosomal localization by FIP3 silencing impairs constitutive CD3ζ phosphorylation and leads to increased total amount of CD3ζ and higher TCR–CD3 cell surface expression (Bouchet et al., 2017). This is consistent with the described effect of Lck-mediated phosphorylation on CD3ζ turnover (D’Oro et al., 2002). Therefore, Lck endosomal localization is key for a variety of Lck functions. Interestingly, Unc119, an adapter protein that activates Rab11 and recruits the actin-based molecular motor myosin 5B, controls Lck traffic in an opposite manner than FIP3. Unc119 also associates to CD3 and CD4 and facilitates Lck activation (Gorska et al., 2004; Gorska et al., 2009). Unc119A cooperates with the ciliary ARL-3 GTPase and its GEF ARL-13B to transfer active Tyr394-phosphorylated Lck to the immunological synapse (Stephen et al., 2018). Lck is associated with membrane rafts (Rodgers and Rose, 1996; Drevot et al., 2002). In this context, Lck localization is also regulated by MAL (Anton et al., 2008, 2011), a small tetraspanin associated with membrane rafts and controlling their polarized intracellular traffic (Martin-Belmonte et al., 2003). Finally, the late endosomal transporter CD222 regulates Lck localization, intracellular traffic, and activation (Pfisterer et al., 2014). Interestingly, these different Lck traffic regulators seem to balance the anterograde (MAL, Unc119, and CD222) and retrograde (FIP3) Lck transport, key to regulate Lck function in T cell activation.

LAT cycles between the plasma membrane, endosomes, and the Golgi. The intracellular LAT compartment is polarized to the immunological synapse concomitantly with those of CD3ζ and Lck. Particular amino acid residues control LAT association to intracellular vesicle pools and its targeting to the synapse (Bonello et al., 2004). Intracellular LAT contributes to the synapse as a second wave, following the formation of microclusters derived from plasma membrane LAT (Bonello et al., 2004; Balagopalan et al., 2018). Several intracellular traffic regulators control LAT localization. Some are common with CD3ζ, such as flagellar transport proteins (Vivar et al., 2016), or vesicle docking and fusion regulators, such as the SNARE VAMP7 or the calcium sensor synaptotagmin-7 (Larghi et al., 2013; Soares et al., 2013). In addition, LAT undergoes retrograde transport from the plasma membrane and endosomes to the Golgi under the control of the Rab6 GTPase, the tSNARE syntaxin-16, and the golgin GMAP210 (Carpier et al., 2018; Zucchetti et al., 2019), which together facilitate LAT delivery to the immunological synapse and subsequent T cell activation. It is likely that a continuous traffic between the plasma membrane and endosomal and Golgi compartments takes place and is modified upon T cell contact with antigen-presenting cells. However, the spatiotemporal organization, sequence of events, and regulation of these events are still ill defined.

The mechanisms described above are thought to target TCR–CD3 complexes and Lck and LAT signaling molecules to the immunological synapse, fueling the formation of signaling microclusters at the plasma membrane. After their dynamic trip within microclusters, TCRs and some of its proximal signaling molecules may be internalized and either recycled back to the plasma membrane to participate in additional cycles of signaling, restored in the vesicular compartment, or degraded to downregulate TCR signaling. This may be modulated by post-transcriptional modifications, such as phosphorylation and ubiquitination (Cenciarelli et al., 1992; D’Oro et al., 1997; Valitutti et al., 1997; Wang et al., 2001; Bonello et al., 2004; Balagopalan et al., 2007, 2011; Huang et al., 2010; Ivanova and Carpino, 2016). Worth noting, the existence of a transient endosomal/Golgi compartment where signaling may continue has been inferred from the presence of active kinases and phosphorylated signaling molecules associated with intracellular compartments after TCR engagement (Luton et al., 1997; Yudushkin and Vale, 2010; reviewed in Alcover et al., 2018; Saveanu et al., 2019; Evnouchidou et al., 2020).

As described above, Rac1 and Cdc42 GTPases transduce TCR signals driving actin cytoskeleton remodeling during immunological synapse formation. These molecules were shown to be associated with vesicles in other cellular types (Phuyal and Farhan, 2019). Interestingly, we observed that a minor fraction of Rac1 in T cells colocalizes with Rab11^+^ recycling endosomes, whereas most of the Rac1 protein seems to be associated with the plasma membrane or diffused in the cytosol. Interestingly, perturbing recycling endosome dynamics by overexpressing the Rab11 effector FIP3 concentrates Rac1 in pericentrosomal endosomes, whereas FIP3 silencing disperses endosomal Rac1 all over the cytoplasm. Importantly, FIP3 silencing releases the tight control of Rac1 on the actin cytoskeleton, inducing T cell overspreading on stimulatory surfaces (i.e., anti-CD3-coated) or on poly-L-lysine-coated surfaces. Moreover, FIP3-silenced cells form larger and asymmetrical immunological synapses. These shape changes could be due, at least in part, to a reduction of T cell rigidity. Therefore, Rac1 association and traffic *via* Rab11 endosomes is key to balance basal *versus* TCR-stimulated actin cytoskeleton rearrangements, perhaps by the differential compartmentalization of Rac1 and its regulatory molecules, such as the GEFs Vav1 or Tiam1. Finally, Rac1 endosomal traffic is required for the regulation of T cell activation leading to cytokine production (Bouchet et al., 2016, 2018).

Vesicle traffic to the synapse may also be involved in the termination of T cell activation, as the inhibitory receptor CTL4, which competes with CD28 co-stimulatory receptor, is also associated with an endo-lysosomal vesicular compartment, which is released at the synapse in a LYST-regulated manner (Linsley et al., 1996; Shiratori et al., 1997; Barrat et al., 1999; Iida et al., 2000).

Finally, T cells forming immunological synapses produce extracellular microvesicles containing TCRs, CD40L, ICOS, and tetraspanins (Blanchard et al., 2002b; Choudhuri et al., 2014; Saliba et al., 2019), as well as RNA and DNA (Mittelbrunn et al., 2011; Torralba et al., 2018). Extracellular vesicle protein and nucleic acid components undergo a process of molecular sorting, since extracellular vesicles are enriched in some components while lacking others (Villarroya-Beltri et al., 2013; Yanez-Mo et al., 2015; Saliba et al., 2019). They accumulate at the synaptic cleft, by a budding process regulated by ESCRT proteins (Choudhuri et al., 2014), where they may play a dual role: first, to reduce TCR cell surface expression to control T cell activation and second, to contribute to dendritic cell priming and maturation and B cell help. This may occur in two ways, by binding MHC–peptide antigen or stimulatory molecules on antigen-presenting cells, such as CD40 or ICOSL (Saliba et al., 2019), and by fusing and transferring their microRNA or DNA content (Mittelbrunn et al., 2011; Torralba et al., 2018).

Therefore, a complex balance of exchanges between the plasma membrane and intracellular vesicular compartments, involving the TCR, several signaling molecules, and an array of traffic regulatory proteins ensures TCR signal transduction and actin cytoskeleton remodeling. Distinct spatiotemporal localization of these various proteins may ensure the fidelity of TCR triggering and sustained T cell activation. Finally, the production of extracellular vesicles plays a key role on antigen-presenting cells regulation contributing to dendritic cell priming and maturation or B cell help. Importantly, some of these mechanisms may be altered by pathogen infections or specific genetic disorders. For instance, HIV-1 hijacks these processes to ensure viral replication and transmission and escape from the immune system (see “Alterations of T Cell Cytoskeleton and Molecular Traffic in Pathological Settings” section).

### Role of the Cytoskeleton in Signaling to the Nucleus

One of the consequences of antigen stimulation is the nuclear translocation of several transcription factors, such as nuclear factor of activated T cells (NFAT), nuclear factor kappa B (NFkB), and activator protein 1 (AP1), that play a central role in T cell activation, differentiation, and effector functions. Recent work has highlighted the involvement of the cytoskeleton in controlling this step, particularly in the case of NFAT.

The NFAT family of transcription factors encompasses five different members, two of them being expressed in T cells: NFAT1 (NFATc2 or NFATp) and NFAT2 (NFATc1 or NFATc) (Muller and Rao, 2010). A third member, NFAT4 (NFATc3 or NFATx), is preferentially expressed in thymocytes (Oukka et al., 1998). The expression of these factors may be differentially regulated: for instance, NFAT1 is constitutively expressed in T cells, whereas NFAT2 is induced upon T cell stimulation (Northrop et al., 1994; Lyakh et al., 1997).

In unstimulated T cells, NFAT transcription factors are phosphorylated on a series of serine residues that expand over the nuclear localization signal. Phosphorylation prevents NFAT nuclear translocation, ensuring cytoplasmic localization in resting T cells. NFAT activation is initiated by TCR-induced PLCγ1-dependent production of IP3 and consequent release of Ca^2+^ from ER stores (reviewed in Hogan et al., 2003). Low Ca^2+^ concentration in the ER lumen triggers the multimerization on ER membranes of the single transmembrane domain protein STIM that contacts the pore-forming ORAI proteins on the plasma membrane. As a result, Ca^2+^ influx from the extracellular space is stimulated (Zhang et al., 2005; Prakriya et al., 2006; Penna et al., 2008). The rise of intracellular Ca^2+^ leads to the rapid activation of the Ser/Thr-specific phosphatase calcineurin that binds to and dephosphorylates cytosolic NFAT proteins, leading to their nuclear import (Hogan et al., 2003). Once in the nucleus, NFAT usually acts together with other transcription factors. For instance, it interacts with AP1, FOXP3, or GATA family members (Macian et al., 2001; Monticelli et al., 2004; Wu et al., 2006) and functionally cooperates with NFkB to regulate the transcription of multiple cytokine genes (e.g., IL-2, IL-4, interferon gamma [IFNγ], and IL-17), transcription factors (e.g., FOXP3), or other receptors (e.g., CD25 and CTLA-4) (Muller and Rao, 2010). Notably, NFAT can also act alone to induce CD8 T cell exhaustion (Martinez et al., 2015).

Inactivation of NFAT and its nuclear export depends on the activity of multiple kinases, such as casein kinase 1 (CK1), glycogen synthase kinase 3 (GSK3), and the dual-specificity tyrosine-phosphorylation-regulated kinase (DYRK), that phosphorylate specific motifs in the conserved N-terminal regulatory region (Okamura et al., 2004; Gwack et al., 2006). These kinases have been found to be constitutively associated with NFAT in a large cytoplasmic RNA–protein scaffold complex, which also contains the GTPase IQGAP and the noncoding RNA *NRON* (Sharma et al., 2011). Dephosphorylation of NFAT requires the dissociation of this complex and results in masking the nuclear export sequence (NES) in NFAT, exposing its nuclear localization sequences (NLS), as well as promoting its transcriptional activity (Okamura et al., 2000).

Once the NLSs are exposed, NFAT may reach nuclear pore complexes by simple diffusion in the cytoplasm, before its import into the nucleus. However, several data suggest a potential implication of the microtubule cytoskeleton in this process. Initial findings in neuroblast cells showed that treatments altering tubulin polymerization, such as decreasing cellular zinc or exposure to colchicine or vinblastine, prevent NFAT transport to the nucleus (Mackenzie and Oteiza, 2007). Further studies revealed that NFAT nuclear translocation depends on importin-β and requires tubulin acetylation (Ishiguro et al., 2011). Interestingly, our recent work (Aguera-Gonzalez et al., 2017) has revealed that endogenous NFATc2 forms discrete clusters juxtaposed to microtubules in unstimulated T cells. These clusters move closer to the immunological synapse surface at early time points after activation and then progressively move to the perinuclear region. Moreover, NFAT clusters progressively move away from microtubules, correlating with NFAT shuttling to the nucleus (Aguera-Gonzalez et al., 2017). Hence, these data suggest that the association of NFAT with the microtubule network could facilitate concentration of this transcription factor around the nucleus and/or its interaction with nuclear pores. In agreement with a functional link between NFAT and the microtubules, we have also observed that knockdown of several proteins that control the appropriate organization of the microtubule network, such as the polarity regulators Apc and Dlg1 and the actin–cytoskeleton linker ezrin, impairs NFAT nuclear translocation and transcriptional activity (Lasserre et al., 2010; Aguera-Gonzalez et al., 2017; Juzans et al., 2020). Altogether, these data underscore the involvement of microtubules in driving NFAT nuclear localization.

The role of actin cytoskeleton in NFAT activation is less clear. Indeed, treatment of cells with actin polymerization inhibitors has been shown to affect the Ca^2+^/NFAT pathway; however, the effects were positive or negative depending on the cell type, dose, and/or stimulation protocol (Rivas et al., 2004; Mackenzie and Oteiza, 2007). This is likely due to the multiple roles of actin that is implicated in regulating T cell/antigen-presenting cell interactions, receptor triggering, and signaling complex dynamics at the immunological synapse (see “T Cell Sensing of Antigen Cues, TCR Triggering, and Immunological Synapse Formation” section). Several regulators of the actin cytoskeleton have been implicated in NFAT activation. These include the actin nucleators WASP and WAVE2 (Silvin et al., 2001; Nolz et al., 2006), the Ser/Thr kinase PAK1 (Yablonski et al., 1998a), the GTPase RhoG (Vigorito et al., 2003), and the GEF SLAT (Becart et al., 2008). However, in most cases, these proteins do not affect directly NFAT but act on upstream signaling proteins and/or Ca^2+^ influx. On the other hand, the aforementioned role of ezrin, which binds to actin and can act in concert with Dlg1 to organize the microtubule network at the immunological synapse (Lasserre et al., 2010), suggests that proper crosstalk between actin and microtubule cytoskeletons is required for NFAT nuclear translocation. Importantly, ezrin and Dlg1 may also control NFAT activation *via* Dlg1 interaction with the p38 MAP kinase, indicating an influence of these cytoskeleton crosstalk regulators in TCR signaling (Round et al., 2007; Lasserre et al., 2010).

It is worth noting that both actin and microtubule cytoskeletons are involved in the reorganization of the ER and mitochondria in activated T cells, which is key to regulate TCR-induced Ca^2+^ signaling (reviewed in Babich and Burkhardt, 2013). As mentioned above, the ER has to move toward the plasma membrane in order to allow the contact between STIM oligomers and ORAI and trigger extracellular Ca^2+^ influx. This movement may directly involve microtubules (Grigoriev et al., 2008). On the other hand, the mitochondria have to be repositioned close to membrane Ca^2+^ channels to buffer Ca^2+^ concentration locally and keep these channels active (Ishii et al., 2006; Baixauli et al., 2011; Quintana et al., 2011; Quintana and Hoth, 2012). Polarization of both ER and mitochondria in this setting depends on the coordinated action of actin and microtubules and associated molecular motors (Babich and Burkhardt, 2013).

Finally, another structural link between the nucleus and immunological synapse modulating T cell functions involves A-type lamins. These proteins that belong to the intermediate filaments family and form the nuclear lamina have been shown to indirectly connect with actin and microtubules and affect T cell activation. Indeed, lamin A defective T cells have impaired actin and microtubule dynamics, altered signaling, and a lower ability to form immunological synapses (Gonzalez-Granado et al., 2014).

## Actin–Microtubule Interplay Shaping T Cell Effector Functions

The cooperation between actin and microtubules is also key for T cell effector functions occurring at the synapse, such as lytic granule release or polarized cytokine secretion.

### Lytic Granule Release

**FIGURE 5:**
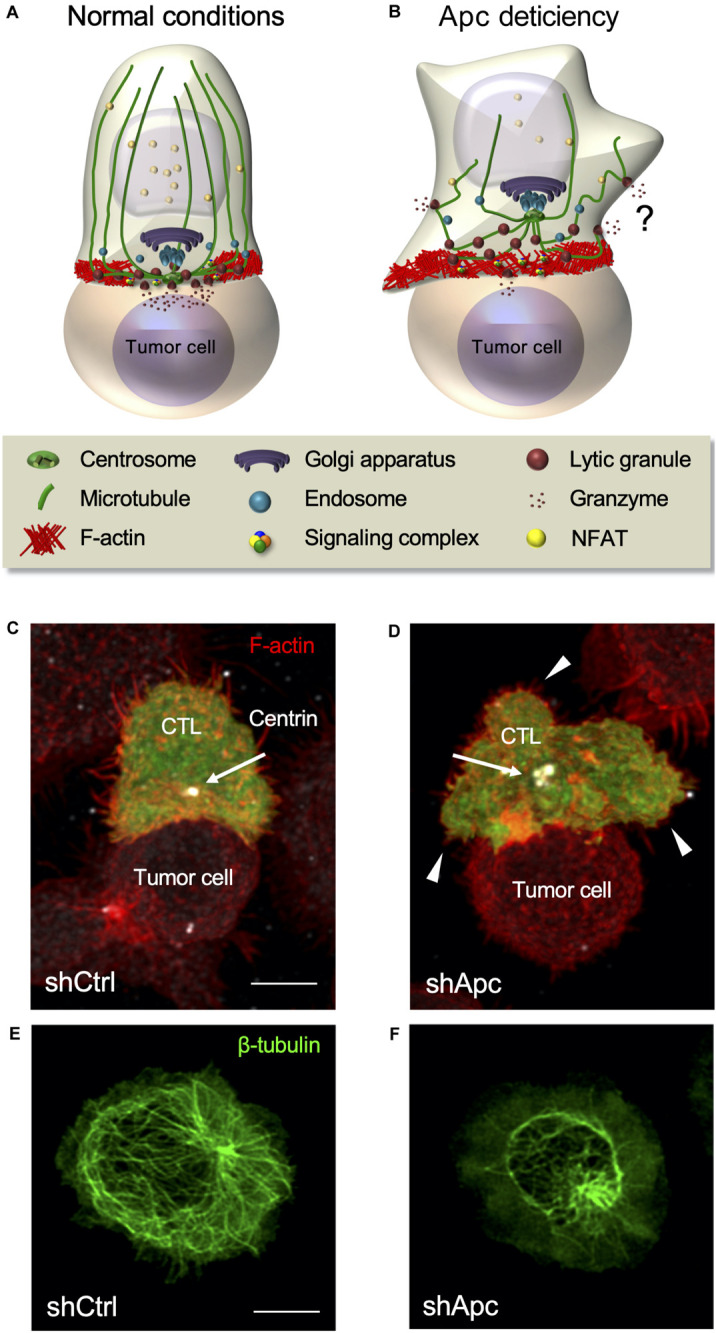
Defects of the polarity regulator and tumor suppressor Apc impair CTL function. **(A,B)** Schematic representation of CTL polarization leading to lytic granule release and killing of a tumor target cell **(A)**. Apc defects impair actin and microtubule reorganization at the immunological synapse, NFAT nuclear translocation, centrosome polarization, immunological synapse symmetry and stability, and lytic granule polarized release leading to tumor target cell killing **(B)**. Granule release is not completely hampered, and it might occur in an unpolarized manner. **(C,D)** Fluorescence confocal microscopy of a control **(C)** or Apc-silenced **(D)** human CTL encountering a tumor target cell coated with an anti-CD3 Ab. Arrows point to the centrosome and arrowheads to large membrane protrusions. Control CTL appears symmetric with the centrosome close to the center of the synapse, whereas Apc-deficient CTL appears dissymmetric with large membrane protrusions and the centrosome distant from the synapse. **(E,F)** Fluorescence confocal microscopy of control **(E)** or Apc-silenced **(F)** human CTLs stimulated anti-CD3-coated coverslips to form immunological pseudo-synapses. Alteration of the microtubule network is evident in Apc-silenced compared with control cells. Confocal images are from Juzans et al. (2020). Bar = 5 μm.

When cytotoxic T cells recognize target cells, lytic granules rapidly move along microtubules, cluster around the moving centrosome, and then polarize with it at the immunological synapse. Centrosome translocation and actin clearance at the synapse have been proposed to be key for lytic granule docking and fusion at the membrane and target cell killing (Stinchcombe et al., 2006; Ritter et al., 2015; Figure 5), although this seems not to be the sole mechanism (Bertrand et al., 2013; Tamzalit et al., 2020). Conversely, actin recovery terminates cytotoxic granule release (Ritter et al., 2017). At the plasma membrane, the centrosome defines a precise secretory domain next to the c-SMAC, concentrating perforin and granzymes in the synaptic cleft (Stinchcombe et al., 2001, 2006). Perforin and granzymes then induce target cell apoptosis. Interestingly, granule movement involves multiple molecular motors. Initially, dynein-dependent retrograde transport on microtubules brings lytic granules to the centrosome (Stinchcombe et al., 2006). Then, granules travel to the immunological synapse, together with the centrosome, and may be positioned close to the plasma membrane by microtubule-based anterograde movement-dependent kinesin motors (Kurowska et al., 2012) or just by the sole proximity of the centrosome to the synapse (Stinchcombe et al., 2006).

The alteration of any of these steps results in impaired lytic granule release. Indeed, the deficiency of several polarity and cytoskeleton regulators impacts both cytoskeleton and centrosome translocation. For instance, silencing of Dlg1 or Apc results in impaired F-actin remodeling, microtubule disorganization, and impaired centrosome and CD3 polarization at the synapse (Round et al., 2005; Lasserre et al., 2010; Humphries et al., 2012; Aguera-Gonzalez et al., 2017; Juzans et al., 2020). Therefore, Dlg1 and Apc modulate CTL immunological synapse formation and function, consequentially influencing both the lytic granule delivery to the synapse and the ability to kill target cells (Silva et al., 2015; Juzans et al., 2020). In addition, the impairment of actin regulators, such as WASP or the Arp2/3 complex, results in altered target cell elimination (De Meester et al., 2010; Randzavola et al., 2019). However, this does not affect lytic granule secretion, assessed by Lamp1 cell surface expression, but impair immunological synapse symmetry and stability (De Meester et al., 2010; Houmadi et al., 2018; Randzavola et al., 2019). Actin dynamics is thus necessary for efficient killing, while apparently not essential for lytic granule release. However, we have recently shown that Lamp1 cell surface measurement could not be sensitive enough to discriminate small secretion differences (Juzans et al., 2020).

Interestingly, several mechanisms of CTL killing may exist, and plasticity could be an attribute of cytotoxic immunological synapses. On the one hand, a mechanism has been described involving centrosome and cytotoxic granule polarization to a well-structured immunological synapse in which actin and microtubule dynamics orchestrate lytic granule delivery to target cells. On the other hand, various examples challenge this rule, questioning the importance of centrosome docking. For instance, the polarity regulator PKCζ is required for centrosome polarization in CD8 T cells, but not for efficient lytic granule release and target cell killing (Ludford-Menting et al., 2005; Bertrand et al., 2013). Its potential role in actin reorganization at the synapse has not been addressed to date, but it has been shown to control F-actin dynamics in migrating T cells (Real et al., 2007; Crespo et al., 2014). Lytic granule translocation to the cytotoxic synapse may occur in the absence of centrosome polarization, and CTLs may simultaneously kill several target cells (Wiedemann et al., 2006; Bertrand et al., 2013). Conversely, human B cells, by inducing weak CD2 signaling, may trigger non-polarized granule exocytosis by the CTLs, although the centrosome is at the synapse (Kabanova et al., 2016; Zurli et al., 2020). Finally, centriole deletion has no effect on lytic granule polarized secretion, but reduces killing efficiency by impairing lytic granule biogenesis and actin-induced forces at the synapse (Tamzalit et al., 2020).

Altering cytoskeleton organization and the interplay between cortical actin and microtubules affects synapse symmetry and stability (Ludford-Menting et al., 2005; Lasserre et al., 2010; Juzans et al., 2020). For instance, we have recently shown that Apc silencing results in altered synapse shape, symmetry, and stability (Juzans et al., 2020; Figures 5A–D). Interestingly, cytotoxic synapses may not require to be fully formed and stable to efficiently kill. Indeed, CTLs exhibit low TCR stimulation threshold to induce lytic granule release compared with the one required for efficient TCR signal transduction to the nucleus (Faroudi et al., 2003). Killing may occur with only three TCR–pMHC interactions, whereas stable synapse formation requires at least 10 interactions (Purbhoo et al., 2004). Finally, the formation of a mature synapse with typical SMAC pattern and CD2 enrichment is not always necessary for efficient cytotoxic granule release (Faroudi et al., 2003; Depoil et al., 2005; O’Keefe and Gajewski, 2005). Therefore, synapse stability may not be necessary for killing, but could increase its efficiency. Indeed, lytic granule release has been spatiotemporally correlated with the forces exerted by CTLs against target cell surfaces. These forces, due to the pushing–pulling action of the actin cytoskeleton, increase target cell membrane tension, which in turn enhances the perforin pore-forming activity (Basu et al., 2016; Tamzalit et al., 2019).

Therefore, actin and microtubule cytoskeleton mechanical properties are crucial for efficient target cell killing, but the extracellular environment may also play a key role. Since the conflicting results described above were most (if not all) obtained *in vitro*, it is possible that the *in vivo* requirements for an effective immune response are more stringent. Indeed, *in vivo*, CTLs act in a crowded environment of healthy or pathological tissues that generate strong forces and interact simultaneously with several cells. Detailed analyses *in vivo* will help to better understand cytotoxicity mechanisms in health and diseases (Boulch et al., 2019).

### Cytokine Secretion

As perforin and granzymes, cytokine secretion involves the Golgi apparatus and the transit through secretory vesicles. Both Golgi and vesicles polarize with the centrosome and facilitate cytokine secretion at the immunological synapse, on a time scale of hours rather than minutes as for lytic granules (Kupfer et al., 1991, 1994; Huse et al., 2006). Interestingly, cytokine polarization is under the control of PKCζ, which is not involved in lytic granule secretion (Bertrand et al., 2010, 2013). Additionally, CD4 T cells can release cytokines in a multidirectional manner (Huse et al., 2006). This could facilitate the dispersion of local signals and the recruitment of target cells. Polarized and multidirectional pathways involve different molecular effectors and could depend on the secreted cytokine, e.g., IL-2, IL-10, and IFNγ are released at the synapse, whereas IL-4 and tumor necrosis factor alpha (TNFα) multi-directionally (Huse et al., 2006). However, this distinction may not be strict, since other authors reported polarized release of IL-4 and TNFα (Depoil et al., 2005; Hivroz et al., 2012). Different experimental setups may explain these differences, suggesting that *in vivo*, cytokine polarization may depend on the stimuli.

The importance of actin clearance from the center of the synapse has been much less addressed for cytokine secretion than for lytic granule release. The impairment of actin dynamics and clearance in Cdc42-silenced or WASP-deficient CD4 T cells significantly decreases IFNγ secretion, without altering its production (Morales-Tirado et al., 2004; Chemin et al., 2012). Interestingly, Cdc42 silencing also inhibits TNF secretion. However, in the setup used by the authors, TNFα is polarized at the synapse and does not appear to be secreted in a multidirectional manner (Chemin et al., 2012; Hivroz et al., 2012). As for lytic granule release (Ritter et al., 2015), impaired actin clearance from the secretion site could act as a physical barrier restraining the access of vesicles to the plasma membrane (Chemin et al., 2012). In addition, in WASP-deficient cells, disorganization of the cis-Golgi morphology appears to take place and could contribute to impaired secretion (Morales-Tirado et al., 2004). Interestingly, impaired actin clearance induced by Apc silencing in CTLs that correlates with reduced lytic granule release does not alter IFNγ nor TNF secretion (Juzans et al., 2020). Hence, the effects of Apc silencing on F-actin appear less significant than those of Cdc42 or WASP deficiency, and Apc may be replaced by another polarity regulator.

The microtubule cytoskeleton seems to play a specific role in the polarized secretion of cytokines. Indeed, nocodazole or vinblastine treatment, which impairs microtubule polymerization and centrosome polarization, alters IFNγ and IL-2 concentrations at the synapse and their polarized secretion. These cytokines are then secreted in a multidirectional manner, likely due to Rab relocalization (Huse et al., 2006; Ueda et al., 2015). On the contrary, nocodazole treatment of CD4 T cells has no effect on multidirectional secretion of TNFα (Huse et al., 2006). Therefore, microtubules would be crucial for cytokine-specific targeting at the synapse but not for their release. Importantly, their alteration could reorient cytokine polarized secretion to a multidirectional one (Huse et al., 2006; Ueda et al., 2015).

The expression of a truncated mutant of ezrin lacking F-actin binding domain that inhibits cortical interaction with the plasma membrane and microtubules leads to defective production of IFNγ and IL-2, but not of TNF (Allenspach et al., 2001). This suggests that the microtubule role and their interplay with the actin are more significant for cytokine secretion in a polarized manner. However, little is known on the actin and microtubule cytoskeleton interplay in cytokine secretion.

Similarly, to what has been observed for lytic granule release, cytokine secretion may not require a well-structured immunological synapse. Indeed, IFNγ production is poorly correlated with extensive TCR clustering in CD4 T cells and depends on the stimulation conditions (Blanchard et al., 2004). However, IFNγ production still requires higher antigen stimulation of CTLs than lytic granule release (Valitutti et al., 1996; Faroudi et al., 2003). Moreover, *in vivo* secretion could be less stringent. Indeed, naive CD4 T cells interact successively with several antigen-presenting cells and undergo synapse–kinapse cycles, promoting IL-2 and IFNγ production (Celli et al., 2005; Sims et al., 2007). Therefore, CD4 T cells could form fewer stable synapses than expected. Interactions with several targets may provide signal integration and facilitate amplification of the immune response or target cell elimination. Furthermore, polarized secretion could still induce signal spreading as the immunological synapse does not spatially restrict IFNγ secretion by CTLs, allowing IFNγ bystander activity important to alter tumor environment (Sanderson et al., 2012; Hoekstra et al., 2020; Thibaut et al., 2020).

## Alterations of T Cell Cytoskeleton and Molecular Traffic in Pathological Settings

As mentioned above, infection of T cells by specific pathogens or genetic alterations may result in dysregulation of the cytoskeleton, endosomal trafficking, and/or their crosstalk, thus impairing TCR signaling, T cell activation, and effector functions. Two examples are described below, i.e., HIV-1 infection of T cells and inherited mutations of the Apc gene in familial adenomatous polyposis.

### HIV-1 Subverts the Interplay Between Endosomal Traffic, TCR Signaling, and Actin Cytoskeleton

HIV-1 infects CD4 T cells hijacking T cell physiology to produce new viral particles and spread to other cells. Viral infection eventually leads to chronic infection and the production of viral reservoirs that escape host immune control. HIV-1 encodes several “accessory” proteins mediating the subversion of various cellular processes. Among these proteins, Nef is key for *in vivo* viral replication and AIDS pathogenesis. Nef is expressed soon upon infection and has pleiotropic effects in T cells, modifying the intracellular environment to enhance virus replication, while reducing host immunity (Fackler et al., 2007). Nef expression subverts endosomal traffic, actin cytoskeleton regulators, and T cell signaling effectors in infected T cells. As a consequence, HIV-1 infection: (i) modifies cell surface expression of several T cell molecules, including CD4, CD28, and MHC class I and II (Pereira and Dasilva, 2016); (ii) reduces actin accumulation at the synapse and alters related features, such as T cell shape, membrane protrusions, cell spreading, and T cell motility (Fackler et al., 1999, 2000; Haller et al., 2006; Rauch et al., 2008; Nobile et al., 2010; Stolp et al., 2010, 2012; Lehmann et al., 2011); and (iii) modulates T cell activation by affecting various signaling pathways, including those controlling activation and apoptosis (Fackler et al., 2007; Abraham et al., 2012; Markle et al., 2013).

**FIGURE 6:**
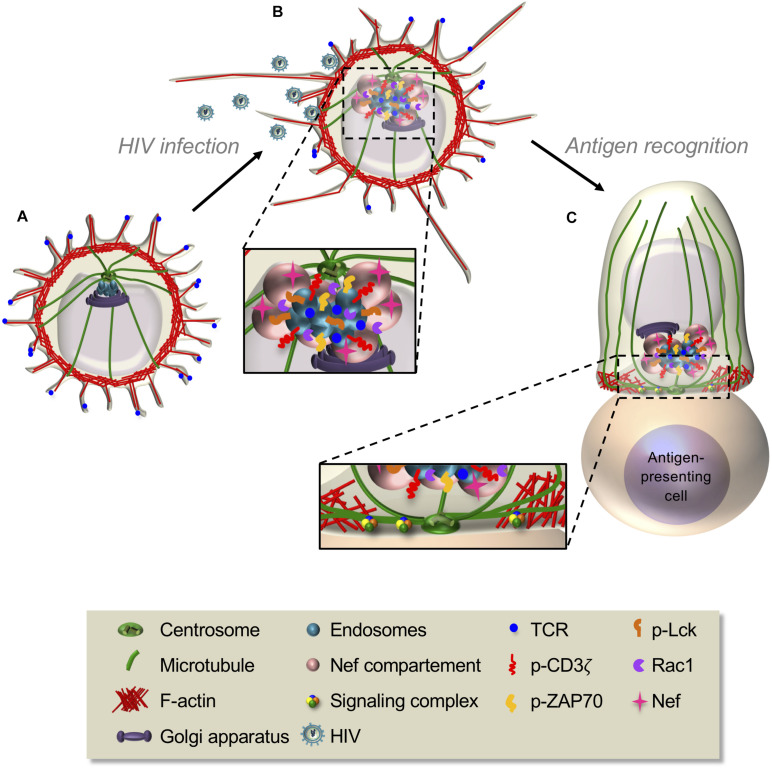
HIV-1 subverts endosomal traffic, TCR signaling, and actin cytoskeleton. **(A,B)** Upon HIV-1 infection, the expression of the viral protein Nef induces pleiotropic effects in the infected T cell, including changes in actin cytoskeleton dynamics and intracellular vesicle traffic. Actin cytoskeleton changes are likely responsible for cell shape modifications (e.g., infected T cells produce less ruffles and longer filopodia), whereas hijacking of endosomal traffic drives changes in the expression of several cell surface molecules and the concentration close to the centrosome of active phosphorylated forms of several proximal TCR signaling molecules, including Lck (p-Lck), the CD3ζ subunit (p-CD3ζ), the tyrosine kinase ZAP-70 (p-ZAP-70), the adapter SLP76, and the cytoskeleton regulators Vav1 and Rac1 (only some depicted here and not depicted in **(A)** because their pattern is diffuse). This Nef-induced “endosomal signaling compartment” (depicted in light blue), which partially overlaps with a larger intracellular compartment containing Nef (in pink), appears to drive the expression of several activation genes, independently of TCR engagement. **(C)** Nef-induced perturbation of signaling molecules and actin cytoskeleton leads to the generation of defective immunological synapses that display fewer signaling complexes and contain a separate endosomal signaling compartment that impairs the TCR signaling cascade (compare with Figure 1E).

The action of Nef on actin cytoskeleton occurs at different levels and appears to affect different stages of the virus life cycle, including virus entry and viral particle assembly, and egress from infected cells and transmission to other cells (Stolp and Fackler, 2011; Bracq et al., 2018). In addition, Nef modifies some intracellular vesicle traffic pathways and as a consequence cellular processes depending on protein transport (Pereira and Dasilva, 2016). Interestingly, Nef perturbs endosomal recycling and hijacks Lck and Rac1 endosomal traffic leading to their concentration in partially overlapping intracellular compartments, thus preventing the formation and signaling function of the immunological synapse (Figure 6; Thoulouze et al., 2006; Del Rio-Iniguez et al., 2018). Nef also limits the communication between LAT and SLP76 adaptors, reducing their capacity to form signaling complexes at the immunological synapse (Abraham et al., 2012). Hence, HIV-1 infection interferes with a key intracellular regulatory hub that ensures the interplay between vesicle traffic, T cell signaling, and actin cytoskeleton remodeling (Bouchet et al., 2016, 2017). Moreover, by concentrating Lck in recycling endosomes, HIV-1 may generate an endosomal signaling compartment, which concentrates Lck in its active form (phosphorylated on Tyr394, see Figure 6), together with tyrosine phosphorylated (i.e., active) species of other signaling molecules, such as CD3ζ, ZAP70, SLP76, and Vav1. In contrast, LAT, associated with different endosomes than Lck, is not concentrated in this compartment. The Nef-induced endosomal compartment likely generates T cell activation signals since a concomitant upregulation of early activation and cytokine genes was observed in the absence of TCR stimulation (Pan et al., 2012; Del Rio-Iniguez et al., 2018). Indeed, impairing the formation of the Nef-induced Lck compartment, by interfering with the endosomal transport regulator FIP3, prevented the upregulation of Nef-induced genes (Del Rio-Iniguez et al., 2018). Interestingly, Nef also extensively sequesters Rac1 in an intracellular compartment partially overlapping with that of Lck. In this manner, Nef modulates Rac1-dependent actin cytoskeleton remodeling and reduces T cell spreading. Thus, by hijacking the endosomal traffic of Lck and Rac1, Nef modulates signaling and actin cytoskeleton-mediated processes in infected T cells (Del Rio-Iniguez et al., 2018).

Therefore, HIV has evolved to subtly modify several regulatory cellular processes at key points of their crosstalk *via* the expression of the viral protein Nef. This may contribute to active steps of virus cycle leading to its replication (Fackler et al., 2007; Pan et al., 2012). It could also be important for inducing latency of infected T cells that favors virus reservoirs and avoids host immune control.

### The Tumor Suppressor Apc in T Cell Physiology and Pathology

Apc is a cell polarity regulator and tumor suppressor whose mutations are associated with familial adenomatous polyposis and colorectal cancer development. Patients suffering from familial polyposis develop hundreds to thousands of polyps in their colon and/or rectum that finally turn into carcinomas if not removed by surgery (Lesko et al., 2014).

Thanks to its multiple binding domains (Figure 2), this large (310-kDa) protein is involved in several cell functions. The central region of Apc contains short peptide motifs that bind the transcriptional co-activator β-catenin and three sets of SAMP (Ser-Ala-Met-Pro) domains that bind Axin to regulate β-catenin (Rubinfeld et al., 1993). Due to its involvement in a protein complex controlling β-catenin degradation, Apc is mostly known for its implication in the Wnt/β-catenin signaling pathway that is essential during embryonic development and crucial for intestinal epithelium homeostasis.

Apc N-terminal portion contains a dimerization domain and an armadillo repeat domain. The latter interacts with many cytoskeleton regulators as the actin and microtubule regulator IQGAP-1, the Rho GTPase Cdc42, the Rho GTPase regulator Asef, or kinesin regulators (Kawasaki et al., 2000; Jimbo et al., 2002; Watanabe et al., 2004; Sudhaharan et al., 2011). The C-terminal portion contains domains binding other cytoskeleton regulators, including the microtubule plus-end binding protein EB1 and Dlg1, but also a basic domain directly interacting with microtubules and modulating their elongation and stability and cell polarity (Munemitsu et al., 1994; Nakamura et al., 2001). This basic domain stimulates F-actin nucleation and filament bundling (Moseley et al., 2007; Okada et al., 2010). Finally, Apc has been shown to interact directly and indirectly with nuclear pore and nuclear transport proteins and apoptosis- or mitosis-related proteins (Nelson and Nathke, 2013).

Although Apc involvement in familial adenomatous polyposis and colorectal cancer has been extensively investigated, most studies concern how and why the epithelium is altered to form premalignant lesions, without questioning if Apc mutations could also alter immunosurveillance processes. Some studies conducted in Apc mutant mice have shown altered control of inflammation by Tregs (Akeus et al., 2014; Chae and Bothwell, 2015). These cells present impaired expression and/or activity of transcription factors key for their effector function regulation, such as FoxP3 and Gata-3, and as a consequence, their differentiation and production of anti-inflammatory cytokines, such as IL-10, are decreased (Gounaris et al., 2009; Aguera-Gonzalez et al., 2017). Interestingly, some studies showed that Apc mutant Tregs start to produce the pro-inflammatory cytokine IL-17 (Gounaris et al., 2009; Chae and Bothwell, 2015), described to promote tumor progression (Chae et al., 2010; Chae and Bothwell, 2015).

Variable alterations of T cell development and survival were observed in mouse models according to the extent and timing of Apc defects. For instance, conditional deletion of Apc in CD4 T cells induced Wnt pathway activation and apoptosis of mature cells leaving the thymus, resulting in lymphopenia (Wong et al., 2015). Likewise, thymocyte-specific Apc loss leads to extensive thymic atrophy due to a blockade of T cell development at the double negative stage (Gounari et al., 2005). Conversely, we observed in Apc^*Min/*+^ mice, bearing a heterozygous mutation in the Apc gene, increased lymphocyte numbers in the spleen and lymph nodes.

Few studies have questioned if Apc loss or mutation directly affects T cell functions at the molecular level. We recently unveiled a direct involvement of Apc in T cell biology and the molecular mechanism responsible for the altered inflammatory control in Apc mutant mice intestine. As mentioned above, Apc loss impairs microtubule organization at the immunological synapse and centrosome reorientation toward the cell contact area in human CD4 T cells (Aguera-Gonzalez et al., 2017). Moreover, we observed that the NFAT transcription factor forms microclusters along microtubules. Therefore, Apc-dependent alteration of the microtubule network impairs NFAT nuclear translocation and its transcriptional activity. Intestinal Tregs from Apc mutant mice appear particularly affected, since they undergo altered differentiation and produce lower amount of the anti-inflammatory cytokine IL-10 (Aguera-Gonzalez et al., 2017), suggesting a dysregulation of the intestinal microenvironment at precancerous stages.

Recently, we showed that Apc is involved in cytoskeleton remodeling at the immunological synapse of CTLs. Indeed, Apc depletion impairs both microtubule and actin cytoskeletons, and as a consequence, it alters the morphology and stability of cytotoxic synapses formed by *ex vivo* differentiated CTLs. Additionally, polarized targeting and dynamics of lytic granules, as well as their fusion at the plasma membrane, are affected, thus diminishing the efficiency of tumor target cell killing by Apc-defective CTLs (Juzans et al., 2020) (see Figure 5). This phenotype shares some similarities with the one of CTLs from Wiscott-Aldrich Syndrome patients, who carry mutations in the gene encoding WASP and present actin cytoskeleton defects (De Meester et al., 2010; Houmadi et al., 2018) reducing their tumor cell killing ability.

Collectively, these data highlight how functional defects of the polarity regulator Apc may have a dual impact on familial adenomatous polyposis and colorectal cancer development, first, by altering the intestinal epithelial homeostasis, and second, by impairing T cell surveillance functions, further favoring the development of precancerous lesions and tumor growth.

## Concluding Remarks

As we reviewed here, the fine interplay between actin and microtubule cytoskeleton and intracellular vesicle traffic is crucial for T cell functions, from migration to TCR signaling, immunological synapse formation, T cell activation, and effector functions. The detailed molecular mechanism of this crosstalk is not fully understood. An array of molecules linking cytoskeletal structures and their regulatory molecules, together with those linking plasma membrane-anchored proteins with the cytoskeleton, is key for this regulation, and their specific action needs further investigation. Likewise, novel cellular features needing cytoskeleton interplay are currently being unveiled. For instance, the role of mechanical forces in T cell physiology is becoming a field of active investigation, and the role of cytoskeletal crosstalk needs its further integration in these processes. *In vivo*, T cells continuously move in a crowded environment from which they may receive mechanical cues. In this sense, intermediate filaments, a third important component of the cell cytoskeleton, appear to play a key role in other cells in ensuring mechanical cell stability, as well as mechano-transduction from the cell surface to the nucleus. Intermediate filament dynamics, function, and interplay with various cell components are still poorly investigated in T cells and will be an interesting field of investigation. Interesting, polarity regulators as Apc ensure the interplay between the three cytoskeletal structures.

## Author Contributions

MM and MJ created the figures. AA and VDB edited the figures. All authors contributed equally in writing and editing the manuscript and read and approved the submitted version.

## Conflict of Interest

The authors declare that the research was conducted in the absence of any commercial or financial relationships that could be construed as a potential conflict of interest.
